# Supplementary material for: LinAge2: providing actionable insights and benchmarking with epigenetic clocks
Source: NPJ Aging. 2025 Apr 23;11(1):29. doi: 10.1038/s41514-025-00221-4 (PMC12019333; doi:10.1038/s41514-025-00221-4)
Supplement: Supplementary file 2 — Supplementary information [file 41514_2025_221_MOESM2_ESM.pdf]

Supplementary Information

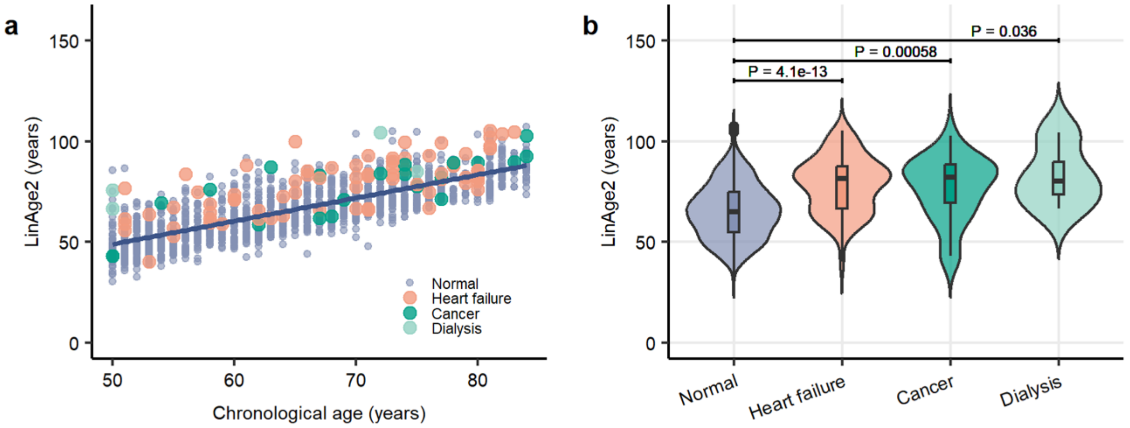

Supplementary Fig. 1: LinAge2 does not lead to excessively high estimates of BA in diseased subjects. a, Scatter plot and linear regression of CA versus LinAge2 in the NHANES IV 1999-2002 waves. Points above the diagonal indicate subjects who were biologically older than their corresponding CA, while points below indicate subjects who were assigned BAs lower than their corresponding CA. LinAge2 is strongly correlated with CA with a regression slope significantly different from zero (Pearson correlation coefficient=0.79, R^2=0.63, P=2.22E-16). Subjects with heart failure were identified using the NHANES variable 'MCQ160B'. Those on long-term dialysis for chronic renal failure were identified using the NHANES variable 'KIQ025'. We also selected subjects who died from cancer within three years based on a combination of NHANES variables as follows: 'MCQ220', 'UCOD_LEADING=2', and 'PERMTH_INT<=36'. Diseased subjects generally appeared to have a higher (above the regression line) BA. Even for subjects with significant disease burden, BA and BA delta never exceeded 105 years and 35 years, respectively. **b,** Violin plots of LinAge2 BAs for the same disease groups compared to all other participants in the 1999-2002 waves. When compared to all other participants (n=4,320), subjects with heart failure (n=74), subjects who died from cancer within three years (n=43), and subjects on long-term dialysis for chronic renal failure (n=12) all exhibited significantly higher biological aging. Groups were compared using Wilcoxon signed-rank tests. Median value, lower (25th) and upper (75th) percentiles are indicated. Lines extend to ±1.5 times interquartile range, with points outside this range drawn individually. The violin shape indicates the probability density function.

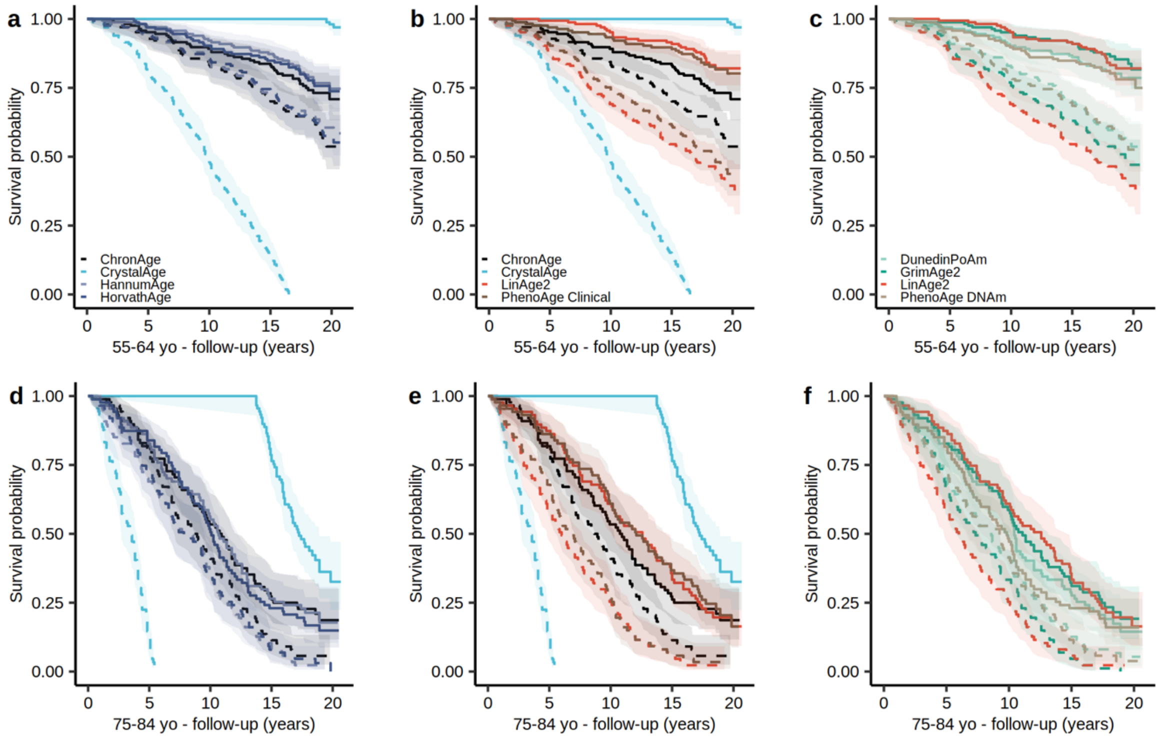

Supplementary Fig. 2: LinAge2 predicts survival in chronologically 55-64 and 75-84 year old individuals. Kaplan-Meier survival curves showing 20-year survival in the **a-c,** 55-64 CA bin (*n*=657) and **d-f,** 75-84 CA bin (*n*=348) in the test cohort. **a,d,** HannumAge, HorvathAge, and ChronAge showed no statistically significant differences in survival. **b,** LinAge2 demonstrated significant survival differences compared to ChronAge in both the best (biologically youngest) 25% (*P*=2.01E-03) and worst (biologically oldest) 25% (*P*=0.03) quartiles. In contrast, PhenoAge Clinical showed a significant difference only in the best 25% quartile (*P*=3.80E-02). **c,f,** In the best 25% quartile, LinAge2 outperformed DunedinPoAm (*P*=6.45E-03 and *P*=1.39E-02 in the 55-64 and 75-84 CA bins, respectively) and PhenoAge DNAm (*P*=8.90E-03 and *P*=1.73E-02 in the 55-64 and 75-84 CA bins, respectively). LinAge2 and GrimAge2 performed similarly with no significant differences between them. In the worst 25% quartile, no significant differences in survival were found between LinAge2, DunedinPoAm, PhenoAge DNAm, and GrimAge2. **e,** Compared to ChronAge, both LinAge2 (*P*=4.56E-03) and PhenoAge Clinical (*P*=3.16E-02) showed significant survival differences in the best 25% quartile, but not in the worst 25% quartile. Clocks were compared using log-rank tests with Benjamini-Hochberg correction. Areas shaded indicate 95% error bands for lines of the same color. yo, years old.

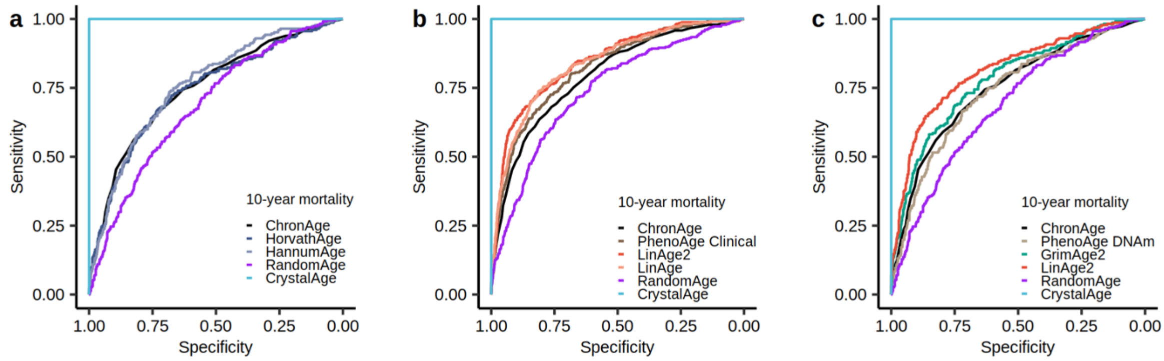

Supplementary Fig. 3: ROC curves for 10-year all-cause mortality in the test cohort. a, There were no significant differences in the AUCs between HorvathAge (AUC=0.7425), HannumAge (AUC=0.7612), and ChronAge (AUC=0.7501) (*n*=1,065). **b,** LinAge2 (AUC=0.8468) was significantly more informative than PhenoAge Clinical (AUC=0.8203, *P*=6.91E-05) and ChronAge (AUC=0.7946, *P*=2.65E-09) in predicting future mortality (*n*=2,036). LinAge2 performed similarly to LinAge (AUC=0.8383). **c,** Compared to LinAge2 (AUC=0.8144), PhenoAge DNAm (AUC=0.7390, *P*=2.84E-06) and GrimAge2 (AUC=0.7801, *P*=1.81E-03) were significantly less predictive of 10-year follow-up (*n*=1,065). Although GrimAge2 outperformed ChronAge (AUC=0.7501, *P*=0.01) in predicting 10-year mortality, PhenoAge DNAm did not (*P*=0.39). ROC curves were compared using DeLong's test.

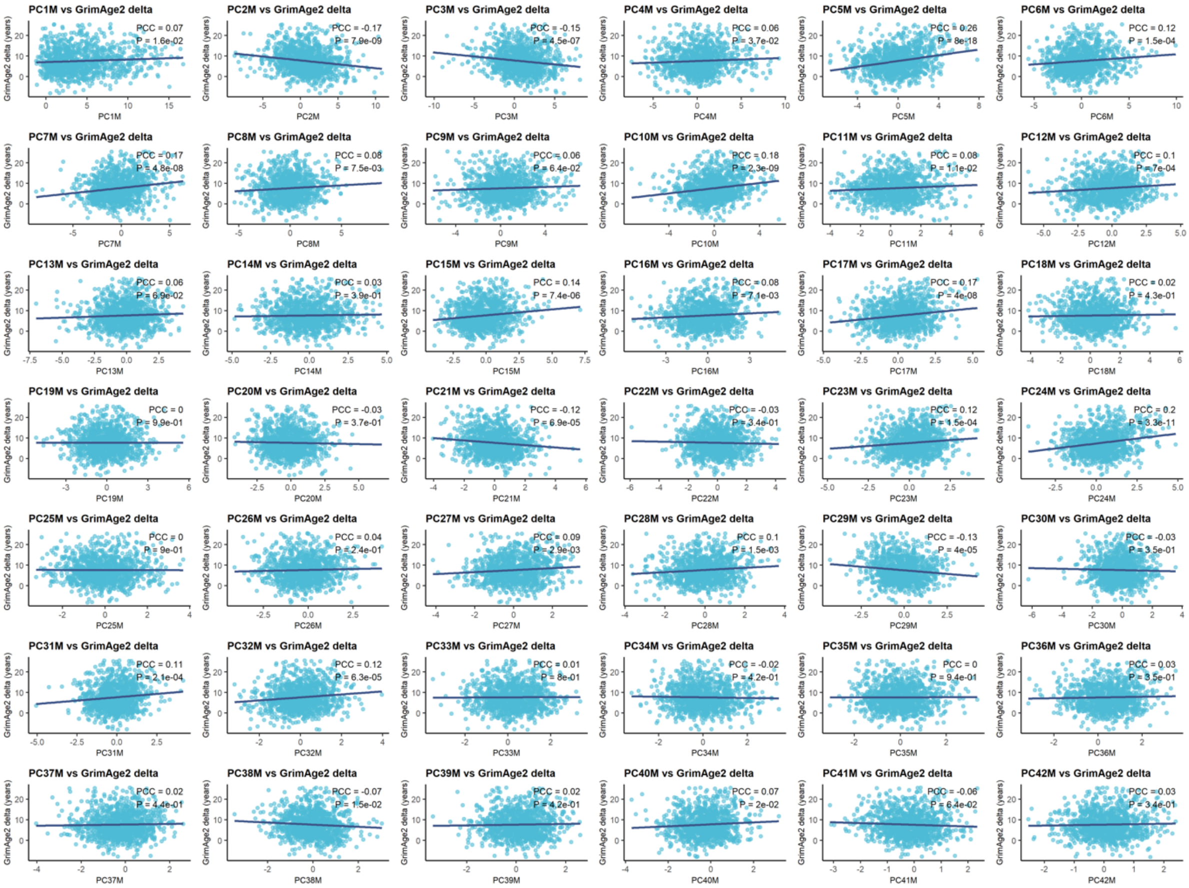

Supplementary Fig. 4: Scatter plots and linear regression of male PCs versus GrimAge2 in the NHANES IV 2001-2002 test wave. Only the first 42 male PCs, which explained 99% of the variance, are shown. Our analysis revealed that a subset of male PCs (PC1M, PC2M, PC5M, PC6M, PC8M, PC11M, PC15M, PC16M, PC17M, PC24M, PC27M, and PC31M) used in constructing LinAge2 showed significant correlations with GrimAge2 age deltas. In contrast, the remaining LinAge2 male PCs (PC19M, PC25M, PC33M, PC36M, and PC42M) did not exhibit significant correlations with GrimAge2 age deltas. Additionally, GrimAge2 age deltas were significantly correlated with a separate set of male PCs not included in the LinAge2 model for males (PC3M, PC4M, PC7M, PC10M, PC12M, PC21M, PC23M, PC28M, PC29M, PC32M, PC38M, and PC40M). PCC, Pearson correlation coefficient. P-value is for the slope of the regression line.

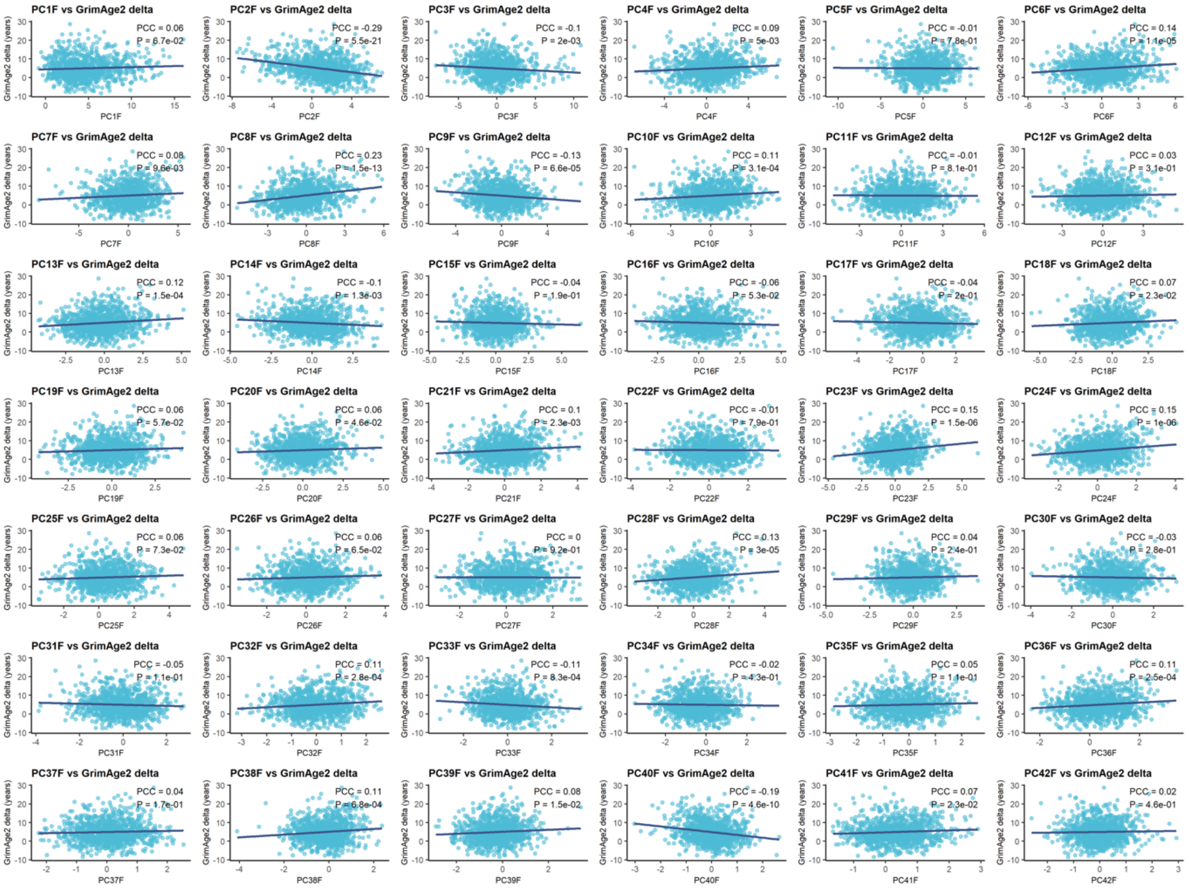

Supplementary Fig. 5: Scatter plots and linear regression of female PCs versus GrimAge2 in the NHANES IV 2001-2002 test wave. Only the first 42 female PCs, which explained 99% of the variance, are shown. Our analysis revealed that a subset of female PCs (PC2F, PC4F, PC6F, PC13F, PC20F, PC23F, PC24F, PC28F, PC32F, PC38F, and PC39F) used in constructing LinAge2 showed significant correlations with GrimAge2 age deltas. In contrast, the remaining LinAge2 female PCs (PC1F, PC11F, PC22F, PC31F, PC35F, and PC37F) did not exhibit significant correlations with GrimAge2 age deltas. Additionally, GrimAge2 age deltas were significantly correlated with a separate set of female PCs not included in the LinAge2 model for females (PC3F, PC7F, PC8F, PC9F, PC10F, PC14F, PC18F, PC21F, PC33F, PC36F, PC40F, and PC41F). PCC, Pearson correlation coefficient. P-value is for the slope of the regression line.

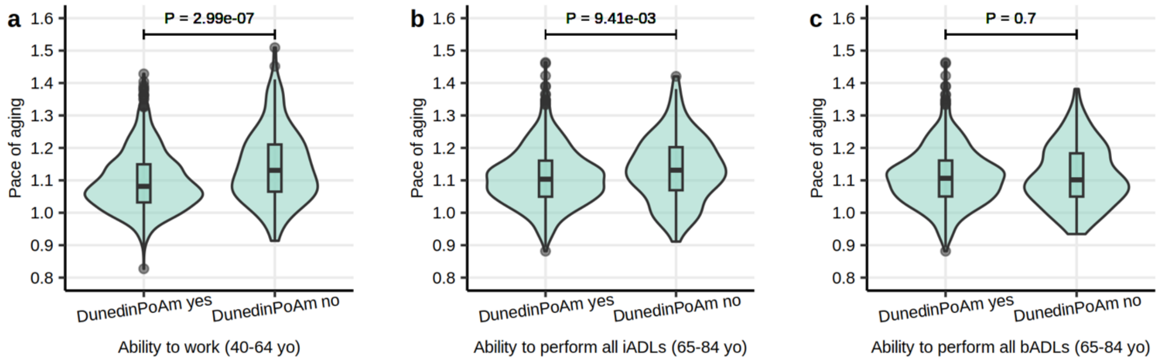

Supplementary Fig. 6: DunedinPoAm tracks with healthspan markers. Violin plots for DunedinPoAm categorized by ability to perform ("yes" group) versus inability to perform ("no" group) **a,** employment work; **b,** instrumental activities of daily living (iADLs); or, **c,** basic activities of daily living (bADLs). Differences in predicted aging rate between higher performers (DunedinPoAm yes) and lower performers (DunedinPoAm no) were significant for a and b, but not for c. Groups were compared using two-sided t-tests. Median value, lower (25th) and upper (75th) percentiles are indicated. Lines extend to $\pm$1.5 times interquartile range, with points outside this range drawn individually. The violin shape indicates the probability density function.

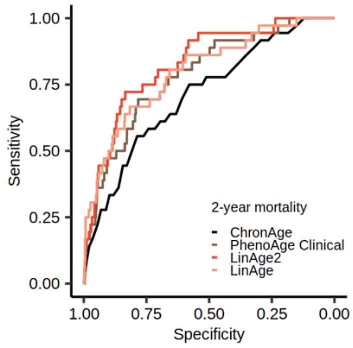

Supplementary Fig. 7: Clinical clocks are better predictors of 2-year mortality than CA. ROC curves for 2-year all-cause mortality in the test cohort. LinAge2 (AUC=0.8294, *P*=4.02E-06), LinAge (AUC=0.8003, *P*=3.91E-04) and PhenoAge Clinical (AUC=0.7870, *P*=3.24E-03) were significantly more predictive of 2-year follow-up than ChronAge (AUC=0.7120) (*n*=2,036). ROC curves were compared using DeLong's test.

Supplementary Table 1. Baseline characteristics of participants.

	**NHANES 1999-2000 (training cohort)**	**NHANES 2001-2002 (testing cohort)**
	***n* = 2,079**	***n* = 2,344**
Age (years) (mean ± SD)	59.85 ± 12.38	58.83 ± 12.56
Male sex (%)	50.26	51.24
Race (%)		
• Non-Hispanic White	47.33	57.59
• Non-Hispanic Black	16.64	17.45
• Mexican American	28.09	18.98
• Other Hispanic	5.48	3.50
• Other	2.45	2.47
Education (%)		
• < High school	43.39	29.96
• High school diploma	20.49	22.92
• > High school	35.93	47.03
• Missing	0.19	0.13
Poverty income ratio (mean ± SD)	2.65 ± 1.60	2.97 ± 1.62
• Missing (%)	14.67	7.23
Smoking (%)		
• Current	18.86	19.84
• No	34.01	33.96
• Missing	47.14	46.20
Alcohol (%)		
• Yes	59.45	61.09
• No	23.18	23.42
• Missing	17.36	15.49
Body mass index (kg/m^2) (mean ± SD)	28.66 ± 5.91	28.77 ± 6.03
Mortality status at 20-year follow-up (%)		
• Alive	55.51	65.02
• Deceased	44.44	34.94
• Missing	0.05	0.04

Supplementary Table 2. Median and median absolute deviation (MAD) values utilized for normalization of clinical parameters, as well as 25th quartile (Q25), 75th quartile (Q75), and individual weights for parameters for LinAge2.

Variable Names	Parameters	Male					Female				
		Median	MAD	Q25	Q75	Individual Weights	Median	MAD	Q25	Q75	Individual Weights
RIDAGEEX	Chronological Age (β_{CA}) (months)	N.A.[#]	N.A.[#]	N.A.[#]	N.A.[#]	-0.0156	N.A.[#]	N.A.[#]	N.A.[#]	N.A.[#]	-0.0092
BMXBMI	Log Body Mass Index (kg/m^2)	3.32	0.17	3.19	3.43	-0.6649	3.35	0.25	3.20	3.54	-0.9209
BPXSAR	Systolic Blood Pressure (mmHg)	122.00	13.34	114.00	131.00	0.6827	118.00	14.83	108.00	130.00	0.3529
BPXDAR	Diastolic Blood Pressure (mmHg)	78.00	10.38	72.00	86.00	-0.6462	75.00	10.38	68.00	82.00	-0.0401
BPXPLS	Pulse Rate (bpm)	70.00	11.86	60.00	78.00	0.6146	72.00	11.86	66.00	80.00	0.7537
LBXHGB	Hemoglobin (g/dL)	15.30	0.89	14.70	16.00	-0.0711	13.50	1.04	12.80	14.22	-0.2515
LBXRBCSI	Red Blood Cell Count (million cells/µL)	5.05	0.37	4.80	5.28	-0.2983	4.43	0.39	4.15	4.67	-0.4521
LBXHCT	Hematocrit (%)	45.50	2.52	43.80	47.40	0.0438	39.70	3.11	37.68	41.90	0.1851
LBXMCVSI	Mean Cell Volume (fL)	90.30	4.45	87.70	93.60	0.5573	90.10	5.04	86.27	93.00	0.9786
LBXMCHSI	Mean Cell Hemoglobin (pg)	30.60	1.78	29.50	31.80	0.2522	30.60	2.08	29.00	31.80	0.3079
LBXMC	Mean Cell Hemoglobin Concentration (g/dL)	33.80	0.74	33.30	34.20	-0.5135	33.85	0.82	33.40	34.40	-1.3520
LBXRDW	Red Cell Distribution Width (%)	12.50	0.59	12.10	12.90	1.2308	12.45	0.82	12.00	13.10	1.0851
LBXPLTSI	Platelet Count (1000 cells/µL)	243.00	48.93	213.00	279.00	-0.2833	277.50	68.20	236.25	326.25	-0.2930
LBXMPSI	Mean Platelet Volume (fL)	8.20	0.74	7.80	8.80	0.1654	8.25	0.82	7.80	8.90	-1.2415
LBXWBCSI	Log White Blood Cell Count (1000 cells/µL)	1.89	0.30	1.70	2.09	0.2960	1.94	0.29	1.74	2.11	0.1576
LBXNEPCT	Segmented Neutrophils Percent (%)	57.40	8.90	51.00	63.30	0.2223	57.50	9.56	51.60	64.32	-0.0336
LBXLYPCT	Lymphocyte Percent (%)	30.60	8.01	25.30	36.30	-0.1679	31.55	8.30	25.48	36.23	0.1625
LBXMOPCT	Monocyte Percent (%)	8.30	2.08	6.90	9.80	-0.2468	7.20	1.78	6.20	8.72	-0.0846
LBXEOPCT	Eosinophils Percent (%)	2.60	1.48	1.70	3.80	-0.0994	2.20	1.33	1.50	3.20	0.0620
LBXBAPCT	Basophils Percent (%)	0.60	0.30	0.40	0.90	0.8416	0.70	0.30	0.48	0.90	-1.1977
LBDNENO	Segmented Neutrophils Number (1000 cells/µL)	3.70	1.33	3.00	4.80	0.7111	4.10	1.63	3.00	5.10	0.1667
LBDLYMNO	Lymphocyte Number (1000 cells/µL)	2.10	0.59	1.70	2.50	0.0369	2.10	0.74	1.70	2.60	0.4609
LBDMONO	Monocyte Number (1000 cells/µL)	0.60	0.15	0.40	0.70	-0.2367	0.50	0.15	0.40	0.60	0.0060
LBDEONO	Eosinophils Number (1000 cells/µL)	0.20	0.15	0.10	0.30	-0.1177	0.10	0.15	0.10	0.20	0.4001
LBDBANO	Basophils Number (1000 cells/µL)	N.A.[¶]	N.A.[¶]	N.A.[¶]	N.A.[¶]	0.0564	N.A.[¶]	N.A.[¶]	N.A.[¶]	N.A.[¶]	-0.0087
LBXCRP	Log C-Reactive Protein (mg/dL)	-1.90	1.13	-2.66	-1.02	-0.0169	-1.27	1.29	-2.12	-0.31	0.3697
LBXSLDSI	Log Lactate Dehydrogenase (U/L)	5.02	0.17	4.91	5.14	-0.5003	4.97	0.19	4.86	5.11	1.2617
LBDIRNSI	Iron (µmol/L)	17.01	6.11	13.25	21.84	0.1572	13.78	5.83	9.85	17.72	0.1110
LBDTIBSI	Total Iron Binding Capacity (µmol/L)	63.19	10.08	56.56	69.99	0.8878	66.86	11.14	59.92	75.59	-0.2346
LBXPCT	Transferrin Saturation (%)	27.90	9.79	21.40	34.70	-0.3471	21.70	9.34	15.28	27.68	0.1989
LBDFERSI	Log Ferritin (µg/L)	5.01	0.77	4.53	5.59	-0.1916	3.69	1.14	2.93	4.46	1.2303
LBDFOLSI	Log Folate (nmol/L)	3.30	0.46	3.01	3.61	0.4887	3.34	0.47	3.03	3.66	0.0644
LBDB12SI	Log Vitamin B12 (pmol/L)	5.93	0.38	5.63	6.14	-0.1330	5.83	0.43	5.54	6.12	-0.2926
LBDSBUSI	Blood Urea Nitrogen (mmol/L)	5.00	1.63	3.90	6.10	0.0037	4.30	1.04	3.60	5.00	1.0587
LBXSNASI	Sodium (mmol/L)	139.50	2.37	138.00	141.10	-0.9851	139.30	2.52	137.48	141.00	0.1037
LBXSKSI	Potassium (mmol/L)	4.17	0.30	3.96	4.35	-0.4695	3.96	0.29	3.81	4.20	-0.1022
LBXSCLSI	Chloride (mmol/L)	102.10	2.97	100.30	104.30	-0.9614	102.80	2.67	100.90	104.40	-0.3935
LBXSC3SI	Bicarbonate (mmol/L)	24.00	1.48	22.00	25.00	0.1890	23.00	1.48	22.00	25.00	1.0227

LBDSCRSI	Creatinine (µmol/L)	70.70	13.05	61.90	79.60	0.0899	53.00	13.05	44.20	61.90	-0.6691
LBDSCASI	Calcium Total (mmol/L)	2.35	0.07	2.30	2.40	1.1378	2.33	0.11	2.25	2.38	-0.6769
LBDSPHSI	Phosphorus (mmol/L)	1.10	0.14	1.00	1.16	0.0550	1.10	0.19	1.00	1.23	0.5391
LBDSTPSI	Protein Total (g/L)	76.00	4.45	73.00	79.00	-0.3174	75.00	4.45	72.00	78.00	-0.4500
LBDSALSI	Albumin (g/L)	46.00	2.97	44.00	47.00	-1.8869	44.00	2.97	42.00	45.00	-0.4568
LBDSGBSI	Globulin (g/L)	30.00	4.45	28.00	33.00	0.9143	31.00	4.45	28.75	34.00	-0.1538
LBDSTBSI	Bilirubin (µmol/L)	10.30	2.52	8.60	13.70	-0.3771	6.80	2.67	5.10	10.30	-0.6931
LBXSAPSI	Log Alkaline Phosphatase (IU/L)	4.37	0.29	4.19	4.57	-0.3960	4.32	0.32	4.06	4.52	1.6302
LBXSATSI	Log Alanine Aminotransferase (U/L)	3.37	0.48	3.09	3.71	-0.4560	2.89	0.37	2.71	3.26	-0.1015
LBXSASSI	Log Aspartate Aminotransferase (U/L)	3.22	0.27	3.04	3.47	0.1985	3.00	0.24	2.83	3.18	0.6217
LBDSUASI	Uric Acid (µmol/L)	339.00	70.57	291.50	392.60	0.9963	255.80	61.82	214.10	303.30	0.7039
LBDSGLSI	Glucose (mmol/L)	5.05	0.58	4.72	5.44	-0.3060	4.88	0.49	4.55	5.22	0.7204
LBXGH	Glycohemoglobin (%)	5.30	0.44	5.10	5.60	0.6176	5.20	0.44	5.00	5.50	0.4117
LDLV	Low-Density Lipoprotein (mmol/L)	3.84	1.05	3.09	4.52	0.1376	3.55	0.96	2.93	4.20	0.5774
SSBNP	Log N-Terminal Pro-Brain Natriuretic Peptide (pg/mL)	3.13	0.91	2.53	3.75	1.9505	3.96	0.79	3.35	4.41	1.9210
URXUMASI	Urine Albumin (mg/L)	7.40	5.78	4.10	12.20	0.6967	7.20	7.49	3.20	15.03	0.0769
URXUCRSI	Log Urine Creatinine (µmol/L)	9.44	0.49	8.99	9.74	0.0999	9.17	0.76	8.48	9.59	-0.1815
crAlbRat	Log Urine Albumin-to-Creatinine Ratio (mg/g)	1.58	0.59	1.23	2.05	0.1664	1.85	0.70	1.44	2.40	0.2231
LBXCOT	Smoking status / Cotinine (ng/mL)	N.A.*	N.A.*	N.A.*	N.A.*	2.9341	N.A.*	N.A.*	N.A.*	N.A.*	3.0916
fs1Score	Co-morbidity index	N.A.[#]	N.A.[#]	N.A.[#]	N.A.[#]	0.0587	N.A.[#]	N.A.[#]	N.A.[#]	N.A.[#]	0.0794
fs2Score	Self-health index	N.A.[#]	N.A.[#]	N.A.[#]	N.A.[#]	0.8806	N.A.[#]	N.A.[#]	N.A.[#]	N.A.[#]	1.0748
fs3Score	Healthcare use index	N.A.[#]	N.A.[#]	N.A.[#]	N.A.[#]	-0.1169	N.A.[#]	N.A.[#]	N.A.[#]	N.A.[#]	0.6267
N.A.[##]	C_0 Constant	N.A.[#]	N.A.[#]	N.A.[#]	N.A.[#]	2.17	N.A.[#]	N.A.[#]	N.A.[#]	N.A.[#]	-3.84

N.A. = not applicable
[¶] N.A. because the median and MAD were 0, hence, actual Basophils Number were used instead
* N.A. because smoking status was determined by using actual serum cotinine levels organized into bins – 0-10 ng/mL (non-smokers), 10-99 ng/mL (light smokers), 100-199 (moderate smokers), and ≥ 200 (heavy smokers) – which could be replaced by questionnaire data if cotinine data are not available
[#] N.A. because actual scores were used
[##] N.A. because the constant does not have a variable name

Supplementary Table 3. PC loadings in LinAge2.

PC (Male)	PC Loadings	Variable Names	Parameters
PC1M	0.463	URXUMASI	Albumin urine (mg/L)
	0.401	crAlbRat (logged)	Log Urine Albumin-to-Creatinine Ratio (mg/g) (logged)
	0.386	fs3Score	Healthcare use index
	0.292	LBXGH	Glycohemoglobin (%)
	0.271	LBDSGLSI	Glucose (mmol/L)
	0.266	SSBNP (logged)	NT-proBNP (pg/ml) (logged)
	0.188	fs2Score	Self-health index
	0.18	BPXSAR	SBP average reported to examinee
	0.152	LBXRDW	Red cell distribution width (percent)
	0.128	LBDSCRSI	Creatinine (umol/L)
	0.125	LBDSBUSI	Blood Urea Nitrogen (mmol/L)
	0.102	LBXCRP (logged)	CRP (mg/dL) (logged)
	0.089	LBDNENO	Segmented neutrophils number (1000 cell/uL)
	0.083	LBXCOT	Cotinine (ng/mL)
	0.063	LBDSGBSI	Globulin (g/L)
	0.061	LBDSUASI	Uric acid (umol/L)
	0.059	LBXEOPCT	Eosinophils percent
	0.053	LBDFOLSI (logged)	Folate serum (nmol/L) (logged)
	0.052	LBXNEPCT	Segmented neutrophils percent
	0.049	LBXMPSI	Mean platelet volume (fL)
	0.042	LBXSLDSI (logged)	Lactate Dehydrogenase (LDH) (U/L) (logged)
	0.033	LBXWBCSI (logged)	WBC count (1000 cells/uL) (logged)
	0.031	LBXMOPCT	Monocyte percent
	0.028	BMXBMI (logged)	Body Mass Index (kg/m2) (logged)
	0.028	LBXMCVSI	Mean cell volume (fL)
	0.027	LBDEONO	Eosinophils number (1000 cells/uL)
	0.025	LBXSAPSI (logged)	Alkaline Phosphatase (ALP) (IU/L) (logged)
	0.025	LBXSKSI	Potassium (mmol/L)
	0.023	LBXBAPCT	Basophils percent
	0.02	fs1Score	Co-morbidity index
	0.019	LBDMONO	Monocyte number (1000 cells/uL)
	0.018	BPXPLS	60 sec pulse (30 sec pulse X2)
	0.011	LBXMCHSI	Mean cell hemoglobin (pg)
	0.009	LBXSC3SI	Bicarbonate (mmol/L)
	0.006	LBDBANO	Basophils number (1000 cells/uL)
	0.004	LBDSTBSI	Bilirubin total (umol/L)
	0.002	LBDSCASI	Calcium total (mmol/L)
	-0.11	LBXHCT	Hematocrit
	-0.104	LBDSALSI	Albumin (g/L)
	-0.1	LBXHGB	Hemoglobin (g/dL)
	-0.099	LBXRBCSI	Red blood cell count (million cells/uL)
	-0.085	BPXDAR	DBP average reported to examinee
	-0.069	URXUCRSI (logged)	Creatinine urine (umol/L) (logged)
	-0.069	LBXLYPCT	Lymphocyte percent
	-0.058	LBXSATSI (logged)	Alanine Aminotransferase (ALT) (U/L) (logged)
	-0.046	LBDLYMNO	Lymphocyte number (1000 cells/uL)
	-0.031	LBDSPHSI	Phosphorus (mmol/L)
	-0.022	LBXPCT	Transferrin Saturation (%)
	-0.022	LBXMC	Mean Cell Hemoglobin Concentration (g/dL)
	-0.022	LBXSCLSI	Chloride (mmol/L)
	-0.021	LBDIRNSI	Iron (umol/L)
	-0.02	LBDB12SI (logged)	Vitamin B12 serum (pmol/L) (logged)
	-0.018	LDLV	Low-Density Lipoprotein (mmol/L)
	-0.017	LBXSASSI (logged)	Aspartate Aminotransferase (AST) (U/L) (logged)
	-0.014	LBDFERSI (logged)	Ferritin (ug/L) (logged)
	-0.01	LBDTIBSI	Total iron binding capacity (umol/L)
	-0.01	LBXSNASI	Sodium (mmol/L)
	-0.007	LBXPLTSI	Platelet count (1000 cells/uL)
	-0.005	LBDSTPSI	Protein total (g/L)

PC2M	0.195	SSBNP (logged)	NT-proBNP (pg/ml) (logged)
	0.177	fs3Score	Healthcare use index
	0.143	LBXRDW	Red cell distribution width (percent)
	0.115	LBDSCRSI	Creatinine (umol/L)
	0.113	LBDSBUSI	Blood Urea Nitrogen (mmol/L)
	0.08	LBXEOPCT	Eosinophils percent
	0.078	LBXMOPCT	Monocyte percent
	0.061	LBXSCLSI	Chloride (mmol/L)
	0.053	LBXSC3SI	Bicarbonate (mmol/L)
	0.048	LBDFOLSI (logged)	Folate serum (nmol/L) (logged)
	0.045	LBXSKSI	Potassium (mmol/L)
	0.036	LBXBAPCT	Basophils percent
	0.019	fs2Score	Self-health index
	0.016	LBXMCVSI	Mean cell volume (fL)
	0.015	LBXSNASI	Sodium (mmol/L)
	0.014	LBDEONO	Eosinophils number (1000 cells/uL)
	0.014	LBDSPHSI	Phosphorus (mmol/L)
	0.006	LBXPLTSI	Platelet count (1000 cells/uL)
	0.006	fs1Score	Co-morbidity index
	0.001	LBDBANO	Basophils number (1000 cells/uL)
	-0.402	LBXHCT	Hematocrit
	-0.397	LBXHGB	Hemoglobin (g/dL)
	-0.312	URXUMASI	Albumin urine (mg/L)
	-0.308	LBXRBCSI	Red blood cell count (million cells/uL)
	-0.215	crAlbRat (logged)	Log Urine Albumin-to-Creatinine Ratio (mg/g) (logged)
	-0.188	LBXGH	Glycohemoglobin (%)
	-0.164	BPXDAR	DBP average reported to examinee
	-0.162	LBXSATSI (logged)	Alanine Aminotransferase (ALT) (U/L) (logged)
	-0.159	LBDSGLSI	Glucose (mmol/L)
	-0.157	LBDFERSI (logged)	Ferritin (ug/L) (logged)
	-0.122	LBDLYMNO	Lymphocyte number (1000 cells/uL)
	-0.12	LBDSTPSI	Protein total (g/L)
	-0.117	LBDIRNSI	Iron (umol/L)
	-0.115	URXUCRSI (logged)	Creatinine urine (umol/L) (logged)
	-0.108	BMXBMI (logged)	Body Mass Index (kg/m2) (logged)
	-0.108	LBXSASSI (logged)	Aspartate Aminotransferase (AST) (U/L) (logged)
	-0.102	LBDSTBSI	Bilirubin total (umol/L)
	-0.099	LBDSALSI	Albumin (g/L)
	-0.096	LBDSCASI	Calcium total (mmol/L)
	-0.095	BPXPLS	60 sec pulse (30 sec pulse X2)
	-0.093	LBXWBCSI (logged)	WBC count (1000 cells/uL) (logged)
	-0.089	LBXPCT	Transferrin Saturation (%)
	-0.077	LBDNENO	Segmented neutrophils number (1000 cell/uL)
	-0.063	LDLV	Low-Density Lipoprotein (mmol/L)
	-0.055	LBXMPSI	Mean platelet volume (fL)
	-0.054	LBDSGBSI	Globulin (g/L)
	-0.052	LBDMONO	Monocyte number (1000 cells/uL)
	-0.047	LBDSUASI	Uric acid (umol/L)
	-0.041	LBDTIBSI	Total iron binding capacity (umol/L)
	-0.041	LBXMC	Mean Cell Hemoglobin Concentration (g/dL)
	-0.035	LBXSAPSI (logged)	Alkaline Phosphatase (ALP) (IU/L) (logged)
	-0.03	LBXLYPCT	Lymphocyte percent
	-0.023	BPXSAR	SBP average reported to examinee
	-0.02	LBDB12SI (logged)	Vitamin B12 serum (pmol/L) (logged)
	-0.019	LBXSLDSI (logged)	Lactate Dehydrogenase (LDH) (U/L) (logged)
	-0.012	LBXCOT	Cotinine (ng/mL)
	-0.005	LBXCRP (logged)	CRP (mg/dL) (logged)
	-0.004	LBXNEPCT	Segmented neutrophils percent
	-0.002	LBXMCHSI	Mean cell hemoglobin (pg)

PC5M	0.407	LBDNENO	Segmented neutrophils number (1000 cell/uL)
	0.302	LBXWBCSI (logged)	WBC count (1000 cells/uL) (logged)
	0.253	LBDMONO	Monocyte number (1000 cells/uL)
	0.242	LBXPLTSI	Platelet count (1000 cells/uL)
	0.241	LBXNEPCT	Segmented neutrophils percent
	0.21	LBXCOT	Cotinine (ng/mL)
	0.188	LBXHGB	Hemoglobin (g/dL)
	0.17	LBXHCT	Hematocrit
	0.157	fs2Score	Self-health index
	0.155	LBXMCVSI	Mean cell volume (fL)
	0.145	LBXMCHSI	Mean cell hemoglobin (pg)
	0.129	SSBNP (logged)	NT-proBNP (pg/ml) (logged)
	0.119	LBXCRP (logged)	CRP (mg/dL) (logged)
	0.106	fs3Score	Healthcare use index
	0.093	LBXSKSI	Potassium (mmol/L)
	0.091	LBDSCASI	Calcium total (mmol/L)
	0.085	BPXPLS	60 sec pulse (30 sec pulse X2)
	0.082	LBDEONO	Eosinophils number (1000 cells/uL)
	0.074	LBDLYMNO	Lymphocyte number (1000 cells/uL)
	0.074	LBXSAPSI (logged)	Alkaline Phosphatase (ALP) (IU/L) (logged)
	0.065	BPXSAR	SBP average reported to examinee
	0.057	LBDTIBSI	Total iron binding capacity (umol/L)
	0.05	LBXMC	Mean Cell Hemoglobin Concentration (g/dL)
	0.044	LBXRDW	Red cell distribution width (percent)
	0.042	LBDFOLSI (logged)	Folate serum (nmol/L) (logged)
	0.04	LBDSPHSI	Phosphorus (mmol/L)
	0.036	LBDSUASI	Uric acid (umol/L)
	0.031	LBDSALSI	Albumin (g/L)
	0.022	LBXRBCSI	Red blood cell count (million cells/uL)
	0.022	LBDSTPSI	Protein total (g/L)
	0.015	LBDSBUSI	Blood Urea Nitrogen (mmol/L)
	0.009	LDLV	Low-Density Lipoprotein (mmol/L)
	0.008	LBDBANO	Basophils number (1000 cells/uL)
	0.006	LBDSCRSI	Creatinine (umol/L)
	0.006	fs1Score	Co-morbidity index
	0.005	LBDSGBSI	Globulin (g/L)
	-0.273	LBDSGLSI	Glucose (mmol/L)
	-0.231	LBXLYPCT	Lymphocyte percent
	-0.204	LBXGH	Glycohemoglobin (%)
	-0.142	URXUCRSI (logged)	Creatinine urine (umol/L) (logged)
	-0.127	URXUMASI	Albumin urine (mg/L)
	-0.122	LBXMPSI	Mean platelet volume (fL)
	-0.116	LBDSTBSI	Bilirubin total (umol/L)
	-0.101	LBXSC3SI	Bicarbonate (mmol/L)
	-0.094	LBXMOPCT	Monocyte percent
	-0.074	LBXPCT	Transferrin Saturation (%)
	-0.059	LBDFERSI (logged)	Ferritin (ug/L) (logged)
	-0.057	BMXBMI (logged)	Body Mass Index (kg/m2) (logged)
	-0.057	LBXSATSI (logged)	Alanine Aminotransferase (ALT) (U/L) (logged)
	-0.055	LBXSASSI (logged)	Aspartate Aminotransferase (AST) (U/L) (logged)
	-0.048	LBXEOPCT	Eosinophils percent
	-0.04	BPXDAR	DBP average reported to examinee
	-0.039	LBDIRNSI	Iron (umol/L)
	-0.039	crAlbRat (logged)	Log Urine Albumin-to-Creatinine Ratio (mg/g) (logged)
	-0.032	LBDB12SI (logged)	Vitamin B12 serum (pmol/L) (logged)
	-0.03	LBXBAPCT	Basophils percent
	-0.025	LBXSCLSI	Chloride (mmol/L)
	-0.023	LBXSLDSI (logged)	Lactate Dehydrogenase (LDH) (U/L) (logged)
	-0.019	LBXSNASI	Sodium (mmol/L)

PC6M	0.349	LBXEOPCT	Eosinophils percent
	0.269	LBDLYMNO	Lymphocyte number (1000 cells/uL)
	0.264	LBXLYPCT	Lymphocyte percent
	0.254	LBDEONO	Eosinophils number (1000 cells/uL)
	0.219	LBXSASSI (logged)	Aspartate Aminotransferase (AST) (U/L) (logged)
	0.207	LBDSGBSI	Globulin (g/L)
	0.197	LBDSTPSI	Protein total (g/L)
	0.181	LBXMOPCT	Monocyte percent
	0.179	LBDMONO	Monocyte number (1000 cells/uL)
	0.176	LBXBAPCT	Basophils percent
	0.149	LBDSCASI	Calcium total (mmol/L)
	0.134	fs3Score	Healthcare use index
	0.127	LBXRDW	Red cell distribution width (percent)
	0.121	LBXCOT	Cotinine (ng/mL)
	0.119	fs2Score	Self-health index
	0.115	LBXSATSI (logged)	Alanine Aminotransferase (ALT) (U/L) (logged)
	0.113	LBDSPHSI	Phosphorus (mmol/L)
	0.092	BPXDAR	DBP average reported to examinee
	0.088	LBXSAPSI (logged)	Alkaline Phosphatase (ALP) (IU/L) (logged)
	0.069	LBXPLTSI	Platelet count (1000 cells/uL)
	0.055	LBXSLDSI (logged)	Lactate Dehydrogenase (LDH) (U/L) (logged)
	0.045	BPXSAR	SBP average reported to examinee
	0.045	LBDTIBSI	Total iron binding capacity (umol/L)
	0.044	LBXMPSI	Mean platelet volume (fL)
	0.037	LBDB12SI (logged)	Vitamin B12 serum (pmol/L) (logged)
	0.035	LBDIRNSI	Iron (umol/L)
	0.029	BPXPLS	60 sec pulse (30 sec pulse X2)
	0.02	LBDFERSI (logged)	Ferritin (ug/L) (logged)
	0.018	LBXPCT	Transferrin Saturation (%)
	0.016	LBXCRP (logged)	CRP (mg/dL) (logged)
	0.016	LBDSUASI	Uric acid (umol/L)
	0.014	crAlbRat (logged)	Log Urine Albumin-to-Creatinine Ratio (mg/g) (logged)
	0.013	LBXGH	Glycohemoglobin (%)
	0.008	LBDBANO	Basophils number (1000 cells/uL)
	0.007	LBXWBCSI (logged)	WBC count (1000 cells/uL) (logged)
	0.006	LBXSKSI	Potassium (mmol/L)
	0.005	fs1Score	Co-morbidity index
	-0.349	LBXNEPCT	Segmented neutrophils percent
	-0.178	LBDSGLSI	Glucose (mmol/L)
	-0.155	LBDNENO	Segmented neutrophils number (1000 cell/uL)
	-0.15	URXUCRSI (logged)	Creatinine urine (umol/L) (logged)
	-0.145	LBXSC3SI	Bicarbonate (mmol/L)
	-0.143	LBXSNASI	Sodium (mmol/L)
	-0.123	LBDSBUSI	Blood Urea Nitrogen (mmol/L)
	-0.101	LBXMC	Mean Cell Hemoglobin Concentration (g/dL)
	-0.099	LBXSCLSI	Chloride (mmol/L)
	-0.082	URXUMASI	Albumin urine (mg/L)
	-0.069	LBDSTBSI	Bilirubin total (umol/L)
	-0.061	LBDSCRSI	Creatinine (umol/L)
	-0.056	LBXHGB	Hemoglobin (g/dL)
	-0.04	LBXMCHSI	Mean cell hemoglobin (pg)
	-0.038	SSBNP (logged)	NT-proBNP (pg/ml) (logged)
	-0.033	LBDFOLSI (logged)	Folate serum (nmol/L) (logged)
	-0.026	LBDSALSI	Albumin (g/L)
	-0.022	LBXHCT	Hematocrit
	-0.021	BMXBMI (logged)	Body Mass Index (kg/m2) (logged)
	-0.007	LBXRBCSI	Red blood cell count (million cells/uL)
	-0.007	LDLV	Low-Density Lipoprotein (mmol/L)
	-0.002	LBXMCVSI	Mean cell volume (fL)

PC8M	0.346	LBXSASSI (logged)	Aspartate Aminotransferase (AST) (U/L) (logged)
	0.26	LBXSLDSI (logged)	Lactate Dehydrogenase (LDH) (U/L) (logged)
	0.226	LBXSATSI (logged)	Alanine Aminotransferase (ALT) (U/L) (logged)
	0.225	LBDSTBSI	Bilirubin total (umol/L)
	0.199	LBDSGBSI	Globulin (g/L)
	0.189	LBDSTPSI	Protein total (g/L)
	0.179	LBXNEPCT	Segmented neutrophils percent
	0.153	LBXRDW	Red cell distribution width (percent)
	0.153	LBDSUASI	Uric acid (umol/L)
	0.126	LBDB12SI (logged)	Vitamin B12 serum (pmol/L) (logged)
	0.122	BPXDAR	DBP average reported to examinee
	0.114	LBDSCASI	Calcium total (mmol/L)
	0.106	LBXSAPSI (logged)	Alkaline Phosphatase (ALP) (IU/L) (logged)
	0.091	BPXPLS	60 sec pulse (30 sec pulse X2)
	0.091	fs2Score	Self-health index
	0.082	LBXCRP (logged)	CRP (mg/dL) (logged)
	0.078	LBDTIBSI	Total iron binding capacity (umol/L)
	0.06	BPXSAR	SBP average reported to examinee
	0.056	fs3Score	Healthcare use index
	0.047	LBDSCRSI	Creatinine (umol/L)
	0.039	LBDSPHSI	Phosphorus (mmol/L)
	0.036	LBDFERSI (logged)	Ferritin (ug/L) (logged)
	0.031	LBDFOLSI (logged)	Folate serum (nmol/L) (logged)
	0.031	LBDNENO	Segmented neutrophils number (1000 cell/uL)
	0.02	BMXBMI (logged)	Body Mass Index (kg/m2) (logged)
	0.004	LBDSBUSI	Blood Urea Nitrogen (mmol/L)
	0.002	SSBNP (logged)	NT-proBNP (pg/ml) (logged)
	0.002	fs1Score	Co-morbidity index
	-0.32	LBXEOPCT	Eosinophils percent
	-0.261	LBDEONO	Eosinophils number (1000 cells/uL)
	-0.2	LBXSNASI	Sodium (mmol/L)
	-0.199	LBXSC3SI	Bicarbonate (mmol/L)
	-0.197	LBDLYMNO	Lymphocyte number (1000 cells/uL)
	-0.18	LBXSCLSI	Chloride (mmol/L)
	-0.155	URXUMASI	Albumin urine (mg/L)
	-0.131	LBXLYPCT	Lymphocyte percent
	-0.12	LBDMONO	Monocyte number (1000 cells/uL)
	-0.109	LBXHGB	Hemoglobin (g/dL)
	-0.1	LBXHCT	Hematocrit
	-0.091	LBXMCVSI	Mean cell volume (fL)
	-0.091	crAlbRat (logged)	Log Urine Albumin-to-Creatinine Ratio (mg/g) (logged)
	-0.088	LBXMCHSI	Mean cell hemoglobin (pg)
	-0.077	LBXWBCSI (logged)	WBC count (1000 cells/uL) (logged)
	-0.077	LBXSKSI	Potassium (mmol/L)
	-0.075	LBXPCT	Transferrin Saturation (%)
	-0.067	LBXBAPCT	Basophils percent
	-0.061	LBXCOT	Cotinine (ng/mL)
	-0.047	LBDIRNSI	Iron (umol/L)
	-0.04	URXUCRSI (logged)	Creatinine urine (umol/L) (logged)
	-0.038	LBXMC	Mean Cell Hemoglobin Concentration (g/dL)
	-0.038	LBXPLTSI	Platelet count (1000 cells/uL)
	-0.029	LDLV	Low-Density Lipoprotein (mmol/L)
	-0.019	LBDSALSI	Albumin (g/L)
	-0.018	LBXMOPCT	Monocyte percent
	-0.018	LBXGH	Glycohemoglobin (%)
	-0.013	LBXRBCSI	Red blood cell count (million cells/uL)
	-0.011	LBDSGLSI	Glucose (mmol/L)
	-0.005	LBDBANO	Basophils number (1000 cells/uL)
	-0.001	LBXMPSI	Mean platelet volume (fL)

PC11M	0.267	LBDSTBSI	Bilirubin total (umol/L)
	0.207	LBXPCT	Transferrin Saturation (%)
	0.205	LBDIRNSI	Iron (umol/L)
	0.19	LBXCOT	Cotinine (ng/mL)
	0.186	LBXRDW	Red cell distribution width (percent)
	0.181	LBDSCASI	Calcium total (mmol/L)
	0.122	LBXPLTSI	Platelet count (1000 cells/uL)
	0.103	fs2Score	Self-health index
	0.101	URXUMASI	Albumin urine (mg/L)
	0.091	LBXLYPCT	Lymphocyte percent
	0.089	LBDSTPSI	Protein total (g/L)
	0.086	crAlbRat (logged)	Log Urine Albumin-to-Creatinine Ratio (mg/g) (logged)
	0.079	LBDSGBSI	Globulin (g/L)
	0.06	LBXSKSI	Potassium (mmol/L)
	0.054	SSBNP (logged)	NT-proBNP (pg/ml) (logged)
	0.037	LBDLYMNO	Lymphocyte number (1000 cells/uL)
	0.034	LBDSPHSI	Phosphorus (mmol/L)
	0.026	LBXRBCSI	Red blood cell count (million cells/uL)
	0.018	LBDSALSI	Albumin (g/L)
	0.009	URXUCRSI (logged)	Creatinine urine (umol/L) (logged)
	-0.269	LBXSLDSI (logged)	Lactate Dehydrogenase (LDH) (U/L) (logged)
	-0.248	LBXSATSI (logged)	Alanine Aminotransferase (ALT) (U/L) (logged)
	-0.246	LBXSASSI (logged)	Aspartate Aminotransferase (AST) (U/L) (logged)
	-0.243	LBXSNASI	Sodium (mmol/L)
	-0.234	LBXSC3SI	Bicarbonate (mmol/L)
	-0.225	BMXBMI (logged)	Body Mass Index (kg/m2) (logged)
	-0.218	BPXDAR	DBP average reported to examinee
	-0.181	BPXSAR	SBP average reported to examinee
	-0.171	LBXEOPCT	Eosinophils percent
	-0.141	LBDSUASI	Uric acid (umol/L)
	-0.138	LBXSCLSI	Chloride (mmol/L)
	-0.136	LBDEONO	Eosinophils number (1000 cells/uL)
	-0.135	fs3Score	Healthcare use index
	-0.117	LBXBAPCT	Basophils percent
	-0.112	LBDFOLSI (logged)	Folate serum (nmol/L) (logged)
	-0.108	LBXCRP (logged)	CRP (mg/dL) (logged)
	-0.106	LBXMPSI	Mean platelet volume (fL)
	-0.101	LBXMC	Mean Cell Hemoglobin Concentration (g/dL)
	-0.1	LBDSBUSI	Blood Urea Nitrogen (mmol/L)
	-0.085	LBDB12SI (logged)	Vitamin B12 serum (pmol/L) (logged)
	-0.085	LBXMCHSI	Mean cell hemoglobin (pg)
	-0.08	LBDMONO	Monocyte number (1000 cells/uL)
	-0.08	LDLV	Low-Density Lipoprotein (mmol/L)
	-0.078	LBDSCRSI	Creatinine (umol/L)
	-0.068	LBXHGB	Hemoglobin (g/dL)
	-0.062	LBXMCVSI	Mean cell volume (fL)
	-0.041	LBXNEPCT	Segmented neutrophils percent
	-0.039	LBXMOPCT	Monocyte percent
	-0.038	LBDNENO	Segmented neutrophils number (1000 cell/uL)
	-0.036	LBXGH	Glycohemoglobin (%)
	-0.034	LBXWBCSI (logged)	WBC count (1000 cells/uL) (logged)
	-0.031	LBXHCT	Hematocrit
	-0.031	LBXSAPSI (logged)	Alkaline Phosphatase (ALP) (IU/L) (logged)
	-0.027	LBDFERSI (logged)	Ferritin (ug/L) (logged)
	-0.025	BPXPLS	60 sec pulse (30 sec pulse X2)
	-0.014	LBDSGLSI	Glucose (mmol/L)
	-0.011	LBDTIBSI	Total iron binding capacity (umol/L)
	-0.005	fs1Score	Co-morbidity index
	-0.003	LBDBANO	Basophils number (1000 cells/uL)

PC15M	0.48	LBXSC3SI	Bicarbonate (mmol/L)
	0.429	fs2Score	Self-health index
	0.204	LBXPCT	Transferrin Saturation (%)
	0.202	LBDFERSI (logged)	Ferritin (ug/L) (logged)
	0.137	URXUCRSI (logged)	Creatinine urine (umol/L) (logged)
	0.137	LBDSCRSI	Creatinine (umol/L)
	0.117	LBXRDW	Red cell distribution width (percent)
	0.117	LBXSAPSI (logged)	Alkaline Phosphatase (ALP) (IU/L) (logged)
	0.115	LBDB12SI (logged)	Vitamin B12 serum (pmol/L) (logged)
	0.11	LBXSLDSI (logged)	Lactate Dehydrogenase (LDH) (U/L) (logged)
	0.108	LBXMPSI	Mean platelet volume (fL)
	0.098	LBDLYMNO	Lymphocyte number (1000 cells/uL)
	0.089	LBDSGBSI	Globulin (g/L)
	0.086	LBDIRNSI	Iron (umol/L)
	0.08	LBXCRP (logged)	CRP (mg/dL) (logged)
	0.071	LBDSUASI	Uric acid (umol/L)
	0.07	LBDSBUSI	Blood Urea Nitrogen (mmol/L)
	0.068	LBXLYPCT	Lymphocyte percent
	0.055	LBXCOT	Cotinine (ng/mL)
	0.049	SSBNP (logged)	NT-proBNP (pg/ml) (logged)
	0.045	LBXWBCSI (logged)	WBC count (1000 cells/uL) (logged)
	0.043	LBDNENO	Segmented neutrophils number (1000 cell/uL)
	0.035	LBDSTPSI	Protein total (g/L)
	0.035	LDLV	Low-Density Lipoprotein (mmol/L)
	0.023	LBXHCT	Hematocrit
	0.018	LBXRBCSI	Red blood cell count (million cells/uL)
	0.01	LBDEONO	Eosinophils number (1000 cells/uL)
	0.004	BPXPLS	60 sec pulse (30 sec pulse X2)
	0.001	LBXMCVSI	Mean cell volume (fL)
	-0.265	LBDTIBSI	Total iron binding capacity (umol/L)
	-0.218	LBDFOLSI (logged)	Folate serum (nmol/L) (logged)
	-0.215	LBDSTBSI	Bilirubin total (umol/L)
	-0.188	LBXSCLSI	Chloride (mmol/L)
	-0.156	LBXBAPCT	Basophils percent
	-0.149	LBXMOPCT	Monocyte percent
	-0.143	fs3Score	Healthcare use index
	-0.142	LBDSCASI	Calcium total (mmol/L)
	-0.101	LBXPLTSI	Platelet count (1000 cells/uL)
	-0.09	LBDMONO	Monocyte number (1000 cells/uL)
	-0.082	LBDSALSI	Albumin (g/L)
	-0.074	LBXGH	Glycohemoglobin (%)
	-0.072	LBXSNASI	Sodium (mmol/L)
	-0.067	crAlbRat (logged)	Log Urine Albumin-to-Creatinine Ratio (mg/g) (logged)
	-0.058	LBDSGLSI	Glucose (mmol/L)
	-0.054	LBXMC	Mean Cell Hemoglobin Concentration (g/dL)
	-0.048	LBXSKSI	Potassium (mmol/L)
	-0.043	BPXDAR	DBP average reported to examinee
	-0.041	URXUMASI	Albumin urine (mg/L)
	-0.04	BMXBMI (logged)	Body Mass Index (kg/m2) (logged)
	-0.029	BPXSAR	SBP average reported to examinee
	-0.025	LBXEOPCT	Eosinophils percent
	-0.021	LBXNEPCT	Segmented neutrophils percent
	-0.018	LBXMCHSI	Mean cell hemoglobin (pg)
	-0.016	LBXSATSI (logged)	Alanine Aminotransferase (ALT) (U/L) (logged)
	-0.007	LBDBANO	Basophils number (1000 cells/uL)
	-0.005	LBXHGB	Hemoglobin (g/dL)
	-0.002	LBXSASSI (logged)	Aspartate Aminotransferase (AST) (U/L) (logged)
	-0.002	fs1Score	Co-morbidity index
	-0.001	LBDSPHSI	Phosphorus (mmol/L)

PC16M	0.306	LBXMOPCT	Monocyte percent
	0.234	LBXSKSI	Potassium (mmol/L)
	0.221	LBXHCT	Hematocrit
	0.219	LBXCOT	Cotinine (ng/mL)
	0.165	BPXSAR	SBP average reported to examinee
	0.149	LBXRDW	Red cell distribution width (percent)
	0.147	SSBNP (logged)	NT-proBNP (pg/ml) (logged)
	0.146	LBXSCLSI	Chloride (mmol/L)
	0.143	LBXHGB	Hemoglobin (g/dL)
	0.119	LBDMONO	Monocyte number (1000 cells/uL)
	0.118	LBXMCVSI	Mean cell volume (fL)
	0.091	LBXRBCSI	Red blood cell count (million cells/uL)
	0.086	LBDSGBSI	Globulin (g/L)
	0.085	LBDSCRSI	Creatinine (umol/L)
	0.08	LBXSNASI	Sodium (mmol/L)
	0.076	LBDB12SI (logged)	Vitamin B12 serum (pmol/L) (logged)
	0.072	LBDFERSI (logged)	Ferritin (ug/L) (logged)
	0.067	LBXMPSI	Mean platelet volume (fL)
	0.066	LBDSCASI	Calcium total (mmol/L)
	0.059	LBXSASSI (logged)	Aspartate Aminotransferase (AST) (U/L) (logged)
	0.059	LBDSGLSI	Glucose (mmol/L)
	0.046	LBXPCT	Transferrin Saturation (%)
	0.044	LBXSAPSI (logged)	Alkaline Phosphatase (ALP) (IU/L) (logged)
	0.028	LBXSLDSI (logged)	Lactate Dehydrogenase (LDH) (U/L) (logged)
	0.025	LBXMCHSI	Mean cell hemoglobin (pg)
	0.023	LBDSTPSI	Protein total (g/L)
	0.019	LBDIRNSI	Iron (umol/L)
	0.018	LBXGH	Glycohemoglobin (%)
	0.01	LBDSBUSI	Blood Urea Nitrogen (mmol/L)
	0.005	LBXLYPCT	Lymphocyte percent
	-0.432	LBDSTBSI	Bilirubin total (umol/L)
	-0.224	LBXEOPCT	Eosinophils percent
	-0.218	fs2Score	Self-health index
	-0.217	LBDEONO	Eosinophils number (1000 cells/uL)
	-0.194	LBXMC	Mean Cell Hemoglobin Concentration (g/dL)
	-0.158	LBXPLTSI	Platelet count (1000 cells/uL)
	-0.156	LBDNENO	Segmented neutrophils number (1000 cell/uL)
	-0.146	LBXWBCSI (logged)	WBC count (1000 cells/uL) (logged)
	-0.132	BMXBMI (logged)	Body Mass Index (kg/m2) (logged)
	-0.124	LBDLYMNO	Lymphocyte number (1000 cells/uL)
	-0.119	LBDSPHSI	Phosphorus (mmol/L)
	-0.113	LBXSC3SI	Bicarbonate (mmol/L)
	-0.102	LBDSALSI	Albumin (g/L)
	-0.085	LBDSUASI	Uric acid (umol/L)
	-0.072	LBDTIBSI	Total iron binding capacity (umol/L)
	-0.071	BPXPLS	60 sec pulse (30 sec pulse X2)
	-0.068	BPXDAR	DBP average reported to examinee
	-0.067	LBDFOLSI (logged)	Folate serum (nmol/L) (logged)
	-0.062	URXUMASI	Albumin urine (mg/L)
	-0.052	LDLV	Low-Density Lipoprotein (mmol/L)
	-0.046	LBXBAPCT	Basophils percent
	-0.041	crAlbRat (logged)	Log Urine Albumin-to-Creatinine Ratio (mg/g) (logged)
	-0.035	LBXNEPCT	Segmented neutrophils percent
	-0.03	LBXSATSI (logged)	Alanine Aminotransferase (ALT) (U/L) (logged)
	-0.026	fs3Score	Healthcare use index
	-0.018	URXUCRSI (logged)	Creatinine urine (umol/L) (logged)
	-0.005	LBDBANO	Basophils number (1000 cells/uL)
	-0.005	fs1Score	Co-morbidity index
	-0.003	LBXCRP (logged)	CRP (mg/dL) (logged)

PC17M	0.243	LBDSGLSI	Glucose (mmol/L)
	0.242	LBXPLTSI	Platelet count (1000 cells/uL)
	0.235	LBXSC3SI	Bicarbonate (mmol/L)
	0.213	URXUCRSI (logged)	Creatinine urine (umol/L) (logged)
	0.201	LBDSUASI	Uric acid (umol/L)
	0.185	BPXSAR	SBP average reported to examinee
	0.184	LBXCOT	Cotinine (ng/mL)
	0.171	LBXBAPCT	Basophils percent
	0.162	BPXDAR	DBP average reported to examinee
	0.135	LBDTIBSI	Total iron binding capacity (umol/L)
	0.133	LBDIRNSI	Iron (umol/L)
	0.108	LBDSCASI	Calcium total (mmol/L)
	0.107	LBXRDW	Red cell distribution width (percent)
	0.097	LBDSGBSI	Globulin (g/L)
	0.086	LBDSTPSI	Protein total (g/L)
	0.08	LBXMCVSI	Mean cell volume (fL)
	0.072	LBXPCT	Transferrin Saturation (%)
	0.072	LBXEOPCT	Eosinophils percent
	0.057	LDLV	Low-Density Lipoprotein (mmol/L)
	0.056	LBXMCHSI	Mean cell hemoglobin (pg)
	0.04	LBXNEPCT	Segmented neutrophils percent
	0.039	LBXSASSI (logged)	Aspartate Aminotransferase (AST) (U/L) (logged)
	0.038	BPXPLS	60 sec pulse (30 sec pulse X2)
	0.035	LBDNENO	Segmented neutrophils number (1000 cell/uL)
	0.034	LBDSCRSI	Creatinine (umol/L)
	0.024	LBDEONO	Eosinophils number (1000 cells/uL)
	0.023	SSBNP (logged)	NT-proBNP (pg/ml) (logged)
	0.017	LBXGH	Glycohemoglobin (%)
	0.017	fs3Score	Healthcare use index
	0.008	LBDBANO	Basophils number (1000 cells/uL)
	0.003	LBXSLDSI (logged)	Lactate Dehydrogenase (LDH) (U/L) (logged)
	-0.455	LBDB12SI (logged)	Vitamin B12 serum (pmol/L) (logged)
	-0.323	LBDFOLSI (logged)	Folate serum (nmol/L) (logged)
	-0.283	fs2Score	Self-health index
	-0.209	crAlbRat (logged)	Log Urine Albumin-to-Creatinine Ratio (mg/g) (logged)
	-0.117	LBDFERSI (logged)	Ferritin (ug/L) (logged)
	-0.109	LBXMPSI	Mean platelet volume (fL)
	-0.094	LBDMONO	Monocyte number (1000 cells/uL)
	-0.09	LBXSCLSI	Chloride (mmol/L)
	-0.079	LBDSPHSI	Phosphorus (mmol/L)
	-0.075	LBXRBCSI	Red blood cell count (million cells/uL)
	-0.058	LBXMOPCT	Monocyte percent
	-0.058	LBXSKSI	Potassium (mmol/L)
	-0.054	LBDLYMNO	Lymphocyte number (1000 cells/uL)
	-0.048	LBDSBUSI	Blood Urea Nitrogen (mmol/L)
	-0.043	LBXLYPCT	Lymphocyte percent
	-0.04	URXUMASI	Albumin urine (mg/L)
	-0.035	LBXHGB	Hemoglobin (g/dL)
	-0.035	LBXHCT	Hematocrit
	-0.028	LBDSTBSI	Bilirubin total (umol/L)
	-0.026	LBXMC	Mean Cell Hemoglobin Concentration (g/dL)
	-0.024	LBXSNASI	Sodium (mmol/L)
	-0.015	BMXBMI (logged)	Body Mass Index (kg/m2) (logged)
	-0.012	LBDSALSI	Albumin (g/L)
	-0.011	LBXWBCSI (logged)	WBC count (1000 cells/uL) (logged)
	-0.008	LBXCRP (logged)	CRP (mg/dL) (logged)
	-0.006	LBXSAPSI (logged)	Alkaline Phosphatase (ALP) (IU/L) (logged)
	-0.005	LBXSATSI (logged)	Alanine Aminotransferase (ALT) (U/L) (logged)
	-0.002	fs1Score	Co-morbidity index

PC19M	0.503	LBXMPSI	Mean platelet volume (fL)
	0.185	LBDSCASI	Calcium total (mmol/L)
	0.142	LBDSTPSI	Protein total (g/L)
	0.116	LBDSALSI	Albumin (g/L)
	0.101	LBDTIBSI	Total iron binding capacity (umol/L)
	0.098	LBXMCHSI	Mean cell hemoglobin (pg)
	0.088	LBXSASSI (logged)	Aspartate Aminotransferase (AST) (U/L) (logged)
	0.086	LBXMCVSI	Mean cell volume (fL)
	0.077	LBXMC	Mean Cell Hemoglobin Concentration (g/dL)
	0.075	LBXNEPCT	Segmented neutrophils percent
	0.075	LBDSPHSI	Phosphorus (mmol/L)
	0.071	crAlbRat (logged)	Log Urine Albumin-to-Creatinine Ratio (mg/g) (logged)
	0.07	LBDEONO	Eosinophils number (1000 cells/uL)
	0.063	LBDSGBSI	Globulin (g/L)
	0.054	LBXEOPCT	Eosinophils percent
	0.052	LBXCOT	Cotinine (ng/mL)
	0.052	LBDNENO	Segmented neutrophils number (1000 cell/uL)
	0.049	LBXRDW	Red cell distribution width (percent)
	0.038	LBXSATSI (logged)	Alanine Aminotransferase (ALT) (U/L) (logged)
	0.037	LBXGH	Glycohemoglobin (%)
	0.035	LBXSNASI	Sodium (mmol/L)
	0.031	SSBNP (logged)	NT-proBNP (pg/ml) (logged)
	0.029	LBXHGB	Hemoglobin (g/dL)
	0.027	LBXWBCSI (logged)	WBC count (1000 cells/uL) (logged)
	0.027	LBXSC3SI	Bicarbonate (mmol/L)
	0.004	LBXHCT	Hematocrit
	0.003	URXUMASI	Albumin urine (mg/L)
	-0.392	LBXPLTSI	Platelet count (1000 cells/uL)
	-0.3	LBDB12SI (logged)	Vitamin B12 serum (pmol/L) (logged)
	-0.25	BPXDAR	DBP average reported to examinee
	-0.172	LBXMOPCT	Monocyte percent
	-0.162	LBDMONO	Monocyte number (1000 cells/uL)
	-0.162	LBXSKSI	Potassium (mmol/L)
	-0.15	LBDSBUSI	Blood Urea Nitrogen (mmol/L)
	-0.147	LBXBAPCT	Basophils percent
	-0.136	LBDSGLSI	Glucose (mmol/L)
	-0.136	LBDSTBSI	Bilirubin total (umol/L)
	-0.134	LBXPCT	Transferrin Saturation (%)
	-0.128	URXUCRSI (logged)	Creatinine urine (umol/L) (logged)
	-0.109	LBDFERSI (logged)	Ferritin (ug/L) (logged)
	-0.106	LDLV	Low-Density Lipoprotein (mmol/L)
	-0.098	LBXCRP (logged)	CRP (mg/dL) (logged)
	-0.091	LBDIRNSI	Iron (umol/L)
	-0.088	BPXSAR	SBP average reported to examinee
	-0.088	fs2Score	Self-health index
	-0.084	LBXSAPSI (logged)	Alkaline Phosphatase (ALP) (IU/L) (logged)
	-0.083	LBXSCLSI	Chloride (mmol/L)
	-0.079	LBDSCRSI	Creatinine (umol/L)
	-0.056	LBDFOLSI (logged)	Folate serum (nmol/L) (logged)
	-0.054	LBXRBCSI	Red blood cell count (million cells/uL)
	-0.048	LBXLYPCT	Lymphocyte percent
	-0.041	BMXBMI (logged)	Body Mass Index (kg/m2) (logged)
	-0.038	LBXSLDSI (logged)	Lactate Dehydrogenase (LDH) (U/L) (logged)
	-0.032	BPXPLS	60 sec pulse (30 sec pulse X2)
	-0.028	LBDLYMNO	Lymphocyte number (1000 cells/uL)
	-0.026	LBDSUASI	Uric acid (umol/L)
	-0.017	fs3Score	Healthcare use index
	-0.005	LBDBANO	Basophils number (1000 cells/uL)
	-0.001	fs1Score	Co-morbidity index

PC24M	0.376	LBXRDW	Red cell distribution width (percent)
	0.36	LBDSPHSI	Phosphorus (mmol/L)
	0.315	LBXCOT	Cotinine (ng/mL)
	0.256	LBXBAPCT	Basophils percent
	0.239	BPXDAR	DBP average reported to examinee
	0.235	LBDSTBSI	Bilirubin total (umol/L)
	0.186	LBDB12SI (logged)	Vitamin B12 serum (pmol/L) (logged)
	0.161	LBXMCVSI	Mean cell volume (fL)
	0.154	LBXCRP (logged)	CRP (mg/dL) (logged)
	0.145	LBXMCHSI	Mean cell hemoglobin (pg)
	0.13	LBXSC3SI	Bicarbonate (mmol/L)
	0.114	LBXGH	Glycohemoglobin (%)
	0.105	LBXSKSI	Potassium (mmol/L)
	0.089	BPXPLS	60 sec pulse (30 sec pulse X2)
	0.087	LBXHGB	Hemoglobin (g/dL)
	0.08	LBXHCT	Hematocrit
	0.057	LBXMPSI	Mean platelet volume (fL)
	0.052	LBDSBUSI	Blood Urea Nitrogen (mmol/L)
	0.051	LBXLYPCT	Lymphocyte percent
	0.044	crAlbRat (logged)	Log Urine Albumin-to-Creatinine Ratio (mg/g) (logged)
	0.028	LBXMC	Mean Cell Hemoglobin Concentration (g/dL)
	0.019	LBDFERSI (logged)	Ferritin (ug/L) (logged)
	0.018	LBDLYMNO	Lymphocyte number (1000 cells/uL)
	0.008	LBDBANO	Basophils number (1000 cells/uL)
	0.007	URXUMASI	Albumin urine (mg/L)
	0.005	BMXBMI (logged)	Body Mass Index (kg/m2) (logged)
	0.002	LBDSCRSI	Creatinine (umol/L)
	-0.18	fs2Score	Self-health index
	-0.179	LBXPCT	Transferrin Saturation (%)
	-0.176	LBDIRNSI	Iron (umol/L)
	-0.167	LBDSTPSI	Protein total (g/L)
	-0.145	LBDMONO	Monocyte number (1000 cells/uL)
	-0.138	LBDSCASI	Calcium total (mmol/L)
	-0.135	LBDSGBSI	Globulin (g/L)
	-0.111	LBXPLTSI	Platelet count (1000 cells/uL)
	-0.098	LBXSCLSI	Chloride (mmol/L)
	-0.089	LBDSGLSI	Glucose (mmol/L)
	-0.085	LBDSUASI	Uric acid (umol/L)
	-0.081	BPXSAR	SBP average reported to examinee
	-0.079	LBXMOPCT	Monocyte percent
	-0.076	LBXSASSI (logged)	Aspartate Aminotransferase (AST) (U/L) (logged)
	-0.074	fs3Score	Healthcare use index
	-0.067	LBDFOLSI (logged)	Folate serum (nmol/L) (logged)
	-0.063	SSBNP (logged)	NT-proBNP (pg/ml) (logged)
	-0.058	LBDSALSI	Albumin (g/L)
	-0.051	URXUCRSI (logged)	Creatinine urine (umol/L) (logged)
	-0.05	LBDEONO	Eosinophils number (1000 cells/uL)
	-0.047	LBXEOPCT	Eosinophils percent
	-0.047	LBXRBCSI	Red blood cell count (million cells/uL)
	-0.047	LBXSLDSI (logged)	Lactate Dehydrogenase (LDH) (U/L) (logged)
	-0.032	LBXWBCSI (logged)	WBC count (1000 cells/uL) (logged)
	-0.031	LBXSATSI (logged)	Alanine Aminotransferase (ALT) (U/L) (logged)
	-0.03	LBDNENO	Segmented neutrophils number (1000 cell/uL)
	-0.028	LBXNEPCT	Segmented neutrophils percent
	-0.024	LBXSNASI	Sodium (mmol/L)
	-0.023	LDLV	Low-Density Lipoprotein (mmol/L)
	-0.015	LBXSAPSI (logged)	Alkaline Phosphatase (ALP) (IU/L) (logged)
	-0.012	LBDTIBSI	Total iron binding capacity (umol/L)
	-0.004	fs1Score	Co-morbidity index

PC25M	0.409	SSBNP (logged)	NT-proBNP (pg/ml) (logged)
	0.313	LBXSKSI	Potassium (mmol/L)
	0.302	LBDFOLSI (logged)	Folate serum (nmol/L) (logged)
	0.278	URXUCRSI (logged)	Creatinine urine (umol/L) (logged)
	0.237	LBXBAPCT	Basophils percent
	0.201	LBXCRP (logged)	CRP (mg/dL) (logged)
	0.153	BMXBMI (logged)	Body Mass Index (kg/m2) (logged)
	0.137	LBDSPHSI	Phosphorus (mmol/L)
	0.124	LBXMPSI	Mean platelet volume (fL)
	0.119	LBDSGBSI	Globulin (g/L)
	0.112	BPXSAR	SBP average reported to examinee
	0.101	LBDSTPSI	Protein total (g/L)
	0.09	LBDFERSI (logged)	Ferritin (ug/L) (logged)
	0.09	LBDSUASI	Uric acid (umol/L)
	0.075	LBXMC	Mean Cell Hemoglobin Concentration (g/dL)
	0.069	LBDLYMNO	Lymphocyte number (1000 cells/uL)
	0.053	LDLV	Low-Density Lipoprotein (mmol/L)
	0.05	LBXLYPCT	Lymphocyte percent
	0.048	URXUMASI	Albumin urine (mg/L)
	0.03	LBXRBCSI	Red blood cell count (million cells/uL)
	0.025	LBXSC3SI	Bicarbonate (mmol/L)
	0.019	LBDSCASI	Calcium total (mmol/L)
	0.016	LBXSAPSI (logged)	Alkaline Phosphatase (ALP) (IU/L) (logged)
	0.014	LBXWBCSI (logged)	WBC count (1000 cells/uL) (logged)
	0.009	LBDBANO	Basophils number (1000 cells/uL)
	0.009	fs2Score	Self-health index
	0.007	LBXHGB	Hemoglobin (g/dL)
	0.006	fs1Score	Co-morbidity index
	0.003	LBDTIBSI	Total iron binding capacity (umol/L)
	-0.279	LBDSCRSI	Creatinine (umol/L)
	-0.263	BPXPLS	60 sec pulse (30 sec pulse X2)
	-0.198	LBXSLDSI (logged)	Lactate Dehydrogenase (LDH) (U/L) (logged)
	-0.163	LBXSNASI	Sodium (mmol/L)
	-0.157	crAlbRat (logged)	Log Urine Albumin-to-Creatinine Ratio (mg/g) (logged)
	-0.15	LBXRDW	Red cell distribution width (percent)
	-0.129	LBXSCLSI	Chloride (mmol/L)
	-0.094	LBXCOT	Cotinine (ng/mL)
	-0.082	fs3Score	Healthcare use index
	-0.077	LBXPCT	Transferrin Saturation (%)
	-0.072	LBDIRNSI	Iron (umol/L)
	-0.068	LBXEOPCT	Eosinophils percent
	-0.067	LBDB12SI (logged)	Vitamin B12 serum (pmol/L) (logged)
	-0.067	LBXMCVSI	Mean cell volume (fL)
	-0.061	LBXMOPCT	Monocyte percent
	-0.057	LBDMONO	Monocyte number (1000 cells/uL)
	-0.057	LBDEONO	Eosinophils number (1000 cells/uL)
	-0.05	LBXSASSI (logged)	Aspartate Aminotransferase (AST) (U/L) (logged)
	-0.05	LBDSTBSI	Bilirubin total (umol/L)
	-0.045	LBXPLTSI	Platelet count (1000 cells/uL)
	-0.042	LBXGH	Glycohemoglobin (%)
	-0.029	LBXMCHSI	Mean cell hemoglobin (pg)
	-0.023	LBDSBUSI	Blood Urea Nitrogen (mmol/L)
	-0.021	LBXNEPCT	Segmented neutrophils percent
	-0.02	LBXHCT	Hematocrit
	-0.017	LBDSALSI	Albumin (g/L)
	-0.012	LBDNENO	Segmented neutrophils number (1000 cell/uL)
	-0.008	LBXSATSI (logged)	Alanine Aminotransferase (ALT) (U/L) (logged)
	-0.008	LBDSGLSI	Glucose (mmol/L)
	-0.006	BPXDAR	DBP average reported to examinee

PC27M	0.43	LBXBAPCT	Basophils percent
	0.273	LBDFOLSI (logged)	Folate serum (nmol/L) (logged)
	0.244	LBDSGLSI	Glucose (mmol/L)
	0.203	LBXCOT	Cotinine (ng/mL)
	0.166	LBDSCRSI	Creatinine (umol/L)
	0.125	LBXMPSI	Mean platelet volume (fL)
	0.12	fs2Score	Self-health index
	0.115	LBDB12SI (logged)	Vitamin B12 serum (pmol/L) (logged)
	0.108	LBXSASSI (logged)	Aspartate Aminotransferase (AST) (U/L) (logged)
	0.099	LBDTIBSI	Total iron binding capacity (umol/L)
	0.098	BPXSAR	SBP average reported to examinee
	0.098	LBDSALSI	Albumin (g/L)
	0.096	LBDSUASI	Uric acid (umol/L)
	0.091	LBDSBUSI	Blood Urea Nitrogen (mmol/L)
	0.079	LBDSTPSI	Protein total (g/L)
	0.078	LBXRDW	Red cell distribution width (percent)
	0.071	LBDMONO	Monocyte number (1000 cells/uL)
	0.058	LBDNENO	Segmented neutrophils number (1000 cell/uL)
	0.051	LBDLYMNO	Lymphocyte number (1000 cells/uL)
	0.048	LBXWBCSI (logged)	WBC count (1000 cells/uL) (logged)
	0.037	URXUMASI	Albumin urine (mg/L)
	0.032	LBXSATSI (logged)	Alanine Aminotransferase (ALT) (U/L) (logged)
	0.03	URXUCRSI (logged)	Creatinine urine (umol/L) (logged)
	0.026	LBXRBCSI	Red blood cell count (million cells/uL)
	0.018	LBXHGB	Hemoglobin (g/dL)
	0.016	LBDBANO	Basophils number (1000 cells/uL)
	0.016	LBXHCT	Hematocrit
	0.014	LBDSGBSI	Globulin (g/L)
	0.011	LBXMOPCT	Monocyte percent
	0.002	LBXMC	Mean Cell Hemoglobin Concentration (g/dL)
	0	LBXLYPCT	Lymphocyte percent
	-0.302	LBXSKSI	Potassium (mmol/L)
	-0.292	LBDSCASI	Calcium total (mmol/L)
	-0.214	BMXBMI (logged)	Body Mass Index (kg/m2) (logged)
	-0.2	LBXGH	Glycohemoglobin (%)
	-0.197	LBDSPHSI	Phosphorus (mmol/L)
	-0.196	BPXDAR	DBP average reported to examinee
	-0.196	fs3Score	Healthcare use index
	-0.19	LBXSLDSI (logged)	Lactate Dehydrogenase (LDH) (U/L) (logged)
	-0.133	LBXCRP (logged)	CRP (mg/dL) (logged)
	-0.125	SSBNP (logged)	NT-proBNP (pg/ml) (logged)
	-0.09	LBDFERSI (logged)	Ferritin (ug/L) (logged)
	-0.063	LBXPCT	Transferrin Saturation (%)
	-0.059	LBXSC3SI	Bicarbonate (mmol/L)
	-0.055	crAlbRat (logged)	Log Urine Albumin-to-Creatinine Ratio (mg/g) (logged)
	-0.054	LBXSCLSI	Chloride (mmol/L)
	-0.049	LBXSAPSI (logged)	Alkaline Phosphatase (ALP) (IU/L) (logged)
	-0.043	LDLV	Low-Density Lipoprotein (mmol/L)
	-0.039	LBXPLTSI	Platelet count (1000 cells/uL)
	-0.038	LBXSNASI	Sodium (mmol/L)
	-0.036	LBXEOPCT	Eosinophils percent
	-0.028	BPXPLS	60 sec pulse (30 sec pulse X2)
	-0.024	LBDEONO	Eosinophils number (1000 cells/uL)
	-0.017	LBXMCVSI	Mean cell volume (fL)
	-0.017	LBDSTBSI	Bilirubin total (umol/L)
	-0.016	LBDIRNSI	Iron (umol/L)
	-0.013	LBXMCHSI	Mean cell hemoglobin (pg)
	-0.007	LBXNEPCT	Segmented neutrophils percent
	-0.004	fs1Score	Co-morbidity index

PC31M	0.521	SSBNP (logged)	NT-proBNP (pg/ml) (logged)
	0.312	LBDB12SI (logged)	Vitamin B12 serum (pmol/L) (logged)
	0.195	LBDTIBSI	Total iron binding capacity (umol/L)
	0.193	LBDSUASI	Uric acid (umol/L)
	0.182	LBXSLDSI (logged)	Lactate Dehydrogenase (LDH) (U/L) (logged)
	0.172	BPXPLS	60 sec pulse (30 sec pulse X2)
	0.171	LBDSCASI	Calcium total (mmol/L)
	0.142	LBXBAPCT	Basophils percent
	0.131	LBDIRNSI	Iron (umol/L)
	0.11	LBXSNASI	Sodium (mmol/L)
	0.099	LBXMC	Mean Cell Hemoglobin Concentration (g/dL)
	0.097	LBXCOT	Cotinine (ng/mL)
	0.085	LBXGH	Glycohemoglobin (%)
	0.062	BPXDAR	DBP average reported to examinee
	0.052	LBXPCT	Transferrin Saturation (%)
	0.052	LBXCRP (logged)	CRP (mg/dL) (logged)
	0.049	LBXRBCSI	Red blood cell count (million cells/uL)
	0.039	LBXHGB	Hemoglobin (g/dL)
	0.035	LBXMOPCT	Monocyte percent
	0.034	URXUCRSI (logged)	Creatinine urine (umol/L) (logged)
	0.032	fs2Score	Self-health index
	0.02	LDLV	Low-Density Lipoprotein (mmol/L)
	0.017	BMXBMI (logged)	Body Mass Index (kg/m2) (logged)
	0.011	LBXLYPCT	Lymphocyte percent
	0.008	LBDSALSI	Albumin (g/L)
	0.007	fs1Score	Co-morbidity index
	0.003	LBDBANO	Basophils number (1000 cells/uL)
	0.001	LBDEONO	Eosinophils number (1000 cells/uL)
	0.001	LBXHCT	Hematocrit
	0.001	LBXSCLSI	Chloride (mmol/L)
	-0.373	LBXSKSI	Potassium (mmol/L)
	-0.255	BPXSAR	SBP average reported to examinee
	-0.191	LBDSBUSI	Blood Urea Nitrogen (mmol/L)
	-0.148	LBXSAPSI (logged)	Alkaline Phosphatase (ALP) (IU/L) (logged)
	-0.12	LBDSTBSI	Bilirubin total (umol/L)
	-0.116	LBDSGBSI	Globulin (g/L)
	-0.109	LBDSTPSI	Protein total (g/L)
	-0.099	LBDFERSI (logged)	Ferritin (ug/L) (logged)
	-0.099	LBXSATSI (logged)	Alanine Aminotransferase (ALT) (U/L) (logged)
	-0.083	LBXSASSI (logged)	Aspartate Aminotransferase (AST) (U/L) (logged)
	-0.08	fs3Score	Healthcare use index
	-0.079	LBDFOLSI (logged)	Folate serum (nmol/L) (logged)
	-0.067	LBDSCRSI	Creatinine (umol/L)
	-0.065	LBXMCVSI	Mean cell volume (fL)
	-0.061	crAlbRat (logged)	Log Urine Albumin-to-Creatinine Ratio (mg/g) (logged)
	-0.056	LBDNENO	Segmented neutrophils number (1000 cell/uL)
	-0.056	LBXRDW	Red cell distribution width (percent)
	-0.051	LBXMPSI	Mean platelet volume (fL)
	-0.045	LBXPLTSI	Platelet count (1000 cells/uL)
	-0.044	LBXSC3SI	Bicarbonate (mmol/L)
	-0.033	LBXWBCSI (logged)	WBC count (1000 cells/uL) (logged)
	-0.033	LBDSPHSI	Phosphorus (mmol/L)
	-0.029	LBXNEPCT	Segmented neutrophils percent
	-0.027	LBDLYMNO	Lymphocyte number (1000 cells/uL)
	-0.025	LBDSGLSI	Glucose (mmol/L)
	-0.015	LBXMCHSI	Mean cell hemoglobin (pg)
	-0.006	LBXEOPCT	Eosinophils percent
	-0.006	LBDMONO	Monocyte number (1000 cells/uL)
	-0.003	URXUMASI	Albumin urine (mg/L)

PC33M	0.411	LBDFOLSI (logged)	Folate serum (nmol/L) (logged)
	0.207	LBXMCVSI	Mean cell volume (fL)
	0.187	LBXCOT	Cotinine (ng/mL)
	0.133	LBXSASSI (logged)	Aspartate Aminotransferase (AST) (U/L) (logged)
	0.116	LBXHCT	Hematocrit
	0.114	LBDSUASI	Uric acid (umol/L)
	0.107	LBXCRP (logged)	CRP (mg/dL) (logged)
	0.105	LBXPLTSI	Platelet count (1000 cells/uL)
	0.104	LBDSCRSI	Creatinine (umol/L)
	0.103	LBDSPHSI	Phosphorus (mmol/L)
	0.091	LDLV	Low-Density Lipoprotein (mmol/L)
	0.084	URXUCRSI (logged)	Creatinine urine (umol/L) (logged)
	0.082	LBXSATSI (logged)	Alanine Aminotransferase (ALT) (U/L) (logged)
	0.081	LBXGH	Glycohemoglobin (%)
	0.078	LBDSCASI	Calcium total (mmol/L)
	0.076	fs2Score	Self-health index
	0.062	LBXEOPCT	Eosinophils percent
	0.051	LBXLYPCT	Lymphocyte percent
	0.046	BPXDAR	DBP average reported to examinee
	0.017	LBDEONO	Eosinophils number (1000 cells/uL)
	0.016	BPXSAR	SBP average reported to examinee
	0.014	SSBNP (logged)	NT-proBNP (pg/ml) (logged)
	0.003	BPXPLS	60 sec pulse (30 sec pulse X2)
	0.003	fs1Score	Co-morbidity index
	0.002	URXUMASI	Albumin urine (mg/L)
	0.001	LBDSGLSI	Glucose (mmol/L)
	0	LBXMCHSI	Mean cell hemoglobin (pg)
	-0.457	LBXMC	Mean Cell Hemoglobin Concentration (g/dL)
	-0.262	LBXBAPCT	Basophils percent
	-0.251	LBDSTPSI	Protein total (g/L)
	-0.186	LBDSALSI	Albumin (g/L)
	-0.172	LBDB12SI (logged)	Vitamin B12 serum (pmol/L) (logged)
	-0.171	LBXSKSI	Potassium (mmol/L)
	-0.156	fs3Score	Healthcare use index
	-0.155	LBDTIBSI	Total iron binding capacity (umol/L)
	-0.153	LBDFERSI (logged)	Ferritin (ug/L) (logged)
	-0.152	LBXRDW	Red cell distribution width (percent)
	-0.125	LBDSBUSI	Blood Urea Nitrogen (mmol/L)
	-0.124	LBDSGBSI	Globulin (g/L)
	-0.115	LBXSAPSI (logged)	Alkaline Phosphatase (ALP) (IU/L) (logged)
	-0.109	LBDMONO	Monocyte number (1000 cells/uL)
	-0.074	LBDIRNSI	Iron (umol/L)
	-0.068	LBXSCLSI	Chloride (mmol/L)
	-0.065	LBXSC3SI	Bicarbonate (mmol/L)
	-0.056	LBXSNASI	Sodium (mmol/L)
	-0.051	LBXRBCSI	Red blood cell count (million cells/uL)
	-0.051	LBXHGB	Hemoglobin (g/dL)
	-0.049	LBXMOPCT	Monocyte percent
	-0.043	LBXWBCSI (logged)	WBC count (1000 cells/uL) (logged)
	-0.041	LBDNENO	Segmented neutrophils number (1000 cell/uL)
	-0.041	LBXMPSI	Mean platelet volume (fL)
	-0.033	LBXNEPCT	Segmented neutrophils percent
	-0.015	BMXBMI (logged)	Body Mass Index (kg/m2) (logged)
	-0.014	LBXSLDSI (logged)	Lactate Dehydrogenase (LDH) (U/L) (logged)
	-0.013	LBXPCT	Transferrin Saturation (%)
	-0.01	LBDBANO	Basophils number (1000 cells/uL)
	-0.008	crAlbRat (logged)	Log Urine Albumin-to-Creatinine Ratio (mg/g) (logged)
	-0.006	LBDLYMNO	Lymphocyte number (1000 cells/uL)
	-0.006	LBDSTBSI	Bilirubin total (umol/L)

PC36M	0.349	LBXMC	Mean Cell Hemoglobin Concentration (g/dL)
	0.277	LBXCOT	Cotinine (ng/mL)
	0.232	LBXGH	Glycohemoglobin (%)
	0.219	LBDSCRSI	Creatinine (umol/L)
	0.201	LDLV	Low-Density Lipoprotein (mmol/L)
	0.173	BPXPLS	60 sec pulse (30 sec pulse X2)
	0.153	LBXSASSI (logged)	Aspartate Aminotransferase (AST) (U/L) (logged)
	0.137	BPXSAR	SBP average reported to examinee
	0.137	LBXSATSI (logged)	Alanine Aminotransferase (ALT) (U/L) (logged)
	0.134	LBXSKSI	Potassium (mmol/L)
	0.115	LBDSGBSI	Globulin (g/L)
	0.112	LBXCRP (logged)	CRP (mg/dL) (logged)
	0.097	LBXSC3SI	Bicarbonate (mmol/L)
	0.048	LBXRBCSI	Red blood cell count (million cells/uL)
	0.042	LBXPCT	Transferrin Saturation (%)
	0.04	LBDIRNSI	Iron (umol/L)
	0.039	LBXMOPCT	Monocyte percent
	0.033	LBDSTBSI	Bilirubin total (umol/L)
	0.032	LBDSCASI	Calcium total (mmol/L)
	0.031	URXUCRSI (logged)	Creatinine urine (umol/L) (logged)
	0.03	BMXBMI (logged)	Body Mass Index (kg/m2) (logged)
	0.023	LBDFOLSI (logged)	Folate serum (nmol/L) (logged)
	0.021	LBDNENO	Segmented neutrophils number (1000 cell/uL)
	0.018	LBXNEPCT	Segmented neutrophils percent
	0.018	fs2Score	Self-health index
	0.015	LBXHGB	Hemoglobin (g/dL)
	0.014	LBXEOPCT	Eosinophils percent
	0.012	LBDB12SI (logged)	Vitamin B12 serum (pmol/L) (logged)
	0.005	LBDMONO	Monocyte number (1000 cells/uL)
	0.004	fs1Score	Co-morbidity index
	0.002	LBDBANO	Basophils number (1000 cells/uL)
	0.001	LBXSCLSI	Chloride (mmol/L)
	-0.269	LBXSAPSI (logged)	Alkaline Phosphatase (ALP) (IU/L) (logged)
	-0.251	LBDSGLSI	Glucose (mmol/L)
	-0.244	LBXSLDSI (logged)	Lactate Dehydrogenase (LDH) (U/L) (logged)
	-0.228	LBXMCVSI	Mean cell volume (fL)
	-0.206	LBDSALSI	Albumin (g/L)
	-0.189	LBXPLTSI	Platelet count (1000 cells/uL)
	-0.171	LBXMPSI	Mean platelet volume (fL)
	-0.161	LBDSPHSI	Phosphorus (mmol/L)
	-0.156	LBDSUASI	Uric acid (umol/L)
	-0.149	LBDFERSI (logged)	Ferritin (ug/L) (logged)
	-0.129	LBXRDW	Red cell distribution width (percent)
	-0.127	LBXHCT	Hematocrit
	-0.119	LBDSBUSI	Blood Urea Nitrogen (mmol/L)
	-0.085	BPXDAR	DBP average reported to examinee
	-0.067	SSBNP (logged)	NT-proBNP (pg/ml) (logged)
	-0.062	LBXMCHSI	Mean cell hemoglobin (pg)
	-0.052	crAlbRat (logged)	Log Urine Albumin-to-Creatinine Ratio (mg/g) (logged)
	-0.046	fs3Score	Healthcare use index
	-0.045	LBXSNASI	Sodium (mmol/L)
	-0.042	LBDLYMNO	Lymphocyte number (1000 cells/uL)
	-0.036	LBDTIBSI	Total iron binding capacity (umol/L)
	-0.028	LBXLYPCT	Lymphocyte percent
	-0.024	LBDSTPSI	Protein total (g/L)
	-0.022	LBXBAPCT	Basophils percent
	-0.005	LBDEONO	Eosinophils number (1000 cells/uL)
	-0.004	URXUMASI	Albumin urine (mg/L)
	-0.004	LBXWBCSI (logged)	WBC count (1000 cells/uL) (logged)

PC42M	0.289	LBDTIBSI	Total iron binding capacity (umol/L)
	0.285	LBDFERSI (logged)	Ferritin (ug/L) (logged)
	0.248	LBDSBUSI	Blood Urea Nitrogen (mmol/L)
	0.185	LBDSCASI	Calcium total (mmol/L)
	0.141	LBDSUASI	Uric acid (umol/L)
	0.141	LBDSGBSI	Globulin (g/L)
	0.094	LBXSC3SI	Bicarbonate (mmol/L)
	0.083	BPXSAR	SBP average reported to examinee
	0.077	LBXCOT	Cotinine (ng/mL)
	0.069	LBDNENO	Segmented neutrophils number (1000 cell/uL)
	0.065	BPXPLS	60 sec pulse (30 sec pulse X2)
	0.065	LBXSCLSI	Chloride (mmol/L)
	0.054	LBXSAPSI (logged)	Alkaline Phosphatase (ALP) (IU/L) (logged)
	0.052	LBXEOPCT	Eosinophils percent
	0.048	LBDB12SI (logged)	Vitamin B12 serum (pmol/L) (logged)
	0.046	LBDFOLSI (logged)	Folate serum (nmol/L) (logged)
	0.035	LBDSTBSI	Bilirubin total (umol/L)
	0.033	LBXSLDSI (logged)	Lactate Dehydrogenase (LDH) (U/L) (logged)
	0.031	URXUMASI	Albumin urine (mg/L)
	0.028	LBXBAPCT	Basophils percent
	0.023	LBXMCVSI	Mean cell volume (fL)
	0.021	fs2Score	Self-health index
	0.02	BMXBMI (logged)	Body Mass Index (kg/m2) (logged)
	0.017	LBDEONO	Eosinophils number (1000 cells/uL)
	0.012	LBXNEPCT	Segmented neutrophils percent
	0.01	LBXWBCSI (logged)	WBC count (1000 cells/uL) (logged)
	0.01	LBXGH	Glycohemoglobin (%)
	0.002	LBDBANO	Basophils number (1000 cells/uL)
	0.001	LBXHCT	Hematocrit
	0	LBDIRNSI	Iron (umol/L)
	-0.514	LBDSALSI	Albumin (g/L)
	-0.429	LBXCRP (logged)	CRP (mg/dL) (logged)
	-0.258	LBDSCRSI	Creatinine (umol/L)
	-0.192	LBDSTPSI	Protein total (g/L)
	-0.151	LBXPCT	Transferrin Saturation (%)
	-0.11	LBDSGLSI	Glucose (mmol/L)
	-0.097	LBDSPHSI	Phosphorus (mmol/L)
	-0.095	fs3Score	Healthcare use index
	-0.09	LBXPLTSI	Platelet count (1000 cells/uL)
	-0.084	SSBNP (logged)	NT-proBNP (pg/ml) (logged)
	-0.074	LBXSNASI	Sodium (mmol/L)
	-0.073	LBXSATSI (logged)	Alanine Aminotransferase (ALT) (U/L) (logged)
	-0.062	BPXDAR	DBP average reported to examinee
	-0.057	LBXMC	Mean Cell Hemoglobin Concentration (g/dL)
	-0.053	LBXSASSI (logged)	Aspartate Aminotransferase (AST) (U/L) (logged)
	-0.046	LBXMPSI	Mean platelet volume (fL)
	-0.044	LBXRDW	Red cell distribution width (percent)
	-0.04	LBXMOPCT	Monocyte percent
	-0.04	LBDMONO	Monocyte number (1000 cells/uL)
	-0.016	URXUCRSI (logged)	Creatinine urine (umol/L) (logged)
	-0.016	LBXHGB	Hemoglobin (g/dL)
	-0.013	LBXLYPCT	Lymphocyte percent
	-0.012	LBXSKSI	Potassium (mmol/L)
	-0.01	LBXRBCSI	Red blood cell count (million cells/uL)
	-0.007	LBDLYMNO	Lymphocyte number (1000 cells/uL)
	-0.007	crAlbRat (logged)	Log Urine Albumin-to-Creatinine Ratio (mg/g) (logged)
	-0.006	LDLV	Low-Density Lipoprotein (mmol/L)
	-0.005	LBXMCHSI	Mean cell hemoglobin (pg)
	-0.002	fs1Score	Co-morbidity index

PC (Female)	PC Loadings	Variable Names	Parameters
PC1F	0.479	fs3Score	Healthcare use index
	0.327	LBXGH	Glycohemoglobin (%)
	0.317	URXUMASI	Albumin urine (mg/L)
	0.314	LBDSGLSI	Glucose (mmol/L)
	0.267	BPXSAR	SBP average reported to examinee
	0.259	crAlbRat (logged)	Log Urine Albumin-to-Creatinine Ratio (mg/g) (logged)
	0.23	LBDSBUSI	Blood Urea Nitrogen (mmol/L)
	0.21	fs2Score	Self-health index
	0.182	SSBNP (logged)	NT-proBNP (pg/ml) (logged)
	0.172	LBDSUASI	Uric acid (umol/L)
	0.128	LBXSC3SI	Bicarbonate (mmol/L)
	0.112	LBDFERSI (logged)	Ferritin (ug/L) (logged)
	0.107	LBDEONO	Eosinophils number (1000 cells/uL)
	0.1	LBXRDW	Red cell distribution width (percent)
	0.096	LBDSCRSI	Creatinine (umol/L)
	0.091	LBDSTBSI	Bilirubin total (umol/L)
	0.089	LBXSLDSI (logged)	Lactate Dehydrogenase (LDH) (U/L) (logged)
	0.085	LBDFOLSI (logged)	Folate serum (nmol/L) (logged)
	0.082	LBXSAPSI (logged)	Alkaline Phosphatase (ALP) (IU/L) (logged)
	0.081	LBXSASSI (logged)	Aspartate Aminotransferase (AST) (U/L) (logged)
	0.079	LBDMONO	Monocyte number (1000 cells/uL)
	0.074	LBXSKSI	Potassium (mmol/L)
	0.064	LBDSGBSI	Globulin (g/L)
	0.063	LBXSATSI (logged)	Alanine Aminotransferase (ALT) (U/L) (logged)
	0.062	LBXCOT	Cotinine (ng/mL)
	0.058	LBXMOPCT	Monocyte percent
	0.056	LDLV	Low-Density Lipoprotein (mmol/L)
	0.055	LBDB12SI (logged)	Vitamin B12 serum (pmol/L) (logged)
	0.053	LBXEOPCT	Eosinophils percent
	0.051	LBDSCASI	Calcium total (mmol/L)
	0.049	LBXCRP (logged)	CRP (mg/dL) (logged)
	0.042	LBXMPSI	Mean platelet volume (fL)
	0.034	LBDNENO	Segmented neutrophils number (1000 cell/uL)
	0.03	LBDLYMNO	Lymphocyte number (1000 cells/uL)
	0.03	LBXHCT	Hematocrit
	0.029	LBDSTPSI	Protein total (g/L)
	0.025	BMXBMI (logged)	Body Mass Index (kg/m2) (logged)
	0.025	fs1Score	Co-morbidity index
	0.024	LBXRBCSI	Red blood cell count (million cells/uL)
	0.023	LBDSPHSI	Phosphorus (mmol/L)
	0.02	LBXWBCSI (logged)	WBC count (1000 cells/uL) (logged)
	0.017	LBXNEPCT	Segmented neutrophils percent
	0.015	BPXPLS	60 sec pulse (30 sec pulse X2)
	0.015	LBXPCT	Transferrin Saturation (%)
	0.014	LBXHGB	Hemoglobin (g/dL)
	0.012	LBDIRNSI	Iron (umol/L)
	0.01	LBXSNASI	Sodium (mmol/L)
	0.008	LBDBANO	Basophils number (1000 cells/uL)
	-0.083	BPXDAR	DBP average reported to examinee
	-0.065	URXUCRSI (logged)	Creatinine urine (umol/L) (logged)
	-0.064	LBXSCLSI	Chloride (mmol/L)
	-0.052	LBDSALSI	Albumin (g/L)
	-0.033	LBDTIBSI	Total iron binding capacity (umol/L)
	-0.023	LBXLYPCT	Lymphocyte percent
	-0.022	LBXMC	Mean Cell Hemoglobin Concentration (g/dL)
	-0.018	LBXMCHSI	Mean cell hemoglobin (pg)
	-0.007	LBXPLTSI	Platelet count (1000 cells/uL)
	-0.004	LBXMCVSI	Mean cell volume (fL)
	-0.001	LBXBAPCT	Basophils percent

PC2F	0.339	fs3Score	Healthcare use index
	0.204	LBDSTBSI	Bilirubin total (umol/L)
	0.191	LBXPCT	Transferrin Saturation (%)
	0.19	LBDIRNSI	Iron (umol/L)
	0.189	LBXSC3SI	Bicarbonate (mmol/L)
	0.185	LBXSASSI (logged)	Aspartate Aminotransferase (AST) (U/L) (logged)
	0.178	LBDFOLSI (logged)	Folate serum (nmol/L) (logged)
	0.171	LBXMCVSI	Mean cell volume (fL)
	0.167	LBXMCHSI	Mean cell hemoglobin (pg)
	0.157	SSBNP (logged)	NT-proBNP (pg/ml) (logged)
	0.114	LBDSBUSI	Blood Urea Nitrogen (mmol/L)
	0.109	LBXMOPCT	Monocyte percent
	0.101	LBXSKSI	Potassium (mmol/L)
	0.094	LBXMC	Mean Cell Hemoglobin Concentration (g/dL)
	0.09	LBDFERSI (logged)	Ferritin (ug/L) (logged)
	0.089	LBDB12SI (logged)	Vitamin B12 serum (pmol/L) (logged)
	0.087	BPXSAR	SBP average reported to examinee
	0.083	LBXSATSI (logged)	Alanine Aminotransferase (ALT) (U/L) (logged)
	0.083	LBXSNASI	Sodium (mmol/L)
	0.078	LBXHGB	Hemoglobin (g/dL)
	0.074	LBDSALSI	Albumin (g/L)
	0.067	LBXSLDSI (logged)	Lactate Dehydrogenase (LDH) (U/L) (logged)
	0.059	LBXEOPCT	Eosinophils percent
	0.058	LBXBAPCT	Basophils percent
	0.055	LBXHCT	Hematocrit
	0.047	LBDSCASI	Calcium total (mmol/L)
	0.045	LBXSCLSI	Chloride (mmol/L)
	0.04	LBDSCRSI	Creatinine (umol/L)
	0.034	LBDEONO	Eosinophils number (1000 cells/uL)
	0.032	LBXCOT	Cotinine (ng/mL)
	0.03	fs2Score	Self-health index
	0.028	LBDSPHSI	Phosphorus (mmol/L)
	0.013	LDLV	Low-Density Lipoprotein (mmol/L)
	0.01	LBXLYPCT	Lymphocyte percent
	0.009	fs1Score	Co-morbidity index
	0.004	LBDBANO	Basophils number (1000 cells/uL)
	-0.338	LBDSGLSI	Glucose (mmol/L)
	-0.302	LBXGH	Glycohemoglobin (%)
	-0.272	URXUMASI	Albumin urine (mg/L)
	-0.154	crAlbRat (logged)	Log Urine Albumin-to-Creatinine Ratio (mg/g) (logged)
	-0.146	LBXWBCSI (logged)	WBC count (1000 cells/uL) (logged)
	-0.135	URXUCRSI (logged)	Creatinine urine (umol/L) (logged)
	-0.133	LBDSGBSI	Globulin (g/L)
	-0.124	BMXBMI (logged)	Body Mass Index (kg/m2) (logged)
	-0.122	LBDNENO	Segmented neutrophils number (1000 cell/uL)
	-0.117	LBXCRP (logged)	CRP (mg/dL) (logged)
	-0.106	LBXRDW	Red cell distribution width (percent)
	-0.104	LBXPLTSI	Platelet count (1000 cells/uL)
	-0.097	BPXPLS	60 sec pulse (30 sec pulse X2)
	-0.094	LBDLYMNO	Lymphocyte number (1000 cells/uL)
	-0.084	LBDSTPSI	Protein total (g/L)
	-0.074	LBXSAPSI (logged)	Alkaline Phosphatase (ALP) (IU/L) (logged)
	-0.067	LBXRBCSI	Red blood cell count (million cells/uL)
	-0.06	LBDMONO	Monocyte number (1000 cells/uL)
	-0.051	BPXDAR	DBP average reported to examinee
	-0.037	LBDTIBSI	Total iron binding capacity (umol/L)
	-0.029	LBXNEPCT	Segmented neutrophils percent
	-0.01	LBXMPSI	Mean platelet volume (fL)
	-0.005	LBDSUASI	Uric acid (umol/L)

PC4F	0.339	fs3Score	Healthcare use index
	0.24	LBXRDW	Red cell distribution width (percent)
	0.197	fs2Score	Self-health index
	0.098	LBDMONO	Monocyte number (1000 cells/uL)
	0.091	LBDEONO	Eosinophils number (1000 cells/uL)
	0.087	LBXGH	Glycohemoglobin (%)
	0.086	LBXMOPCT	Monocyte percent
	0.082	LBXEOPCT	Eosinophils percent
	0.082	LBXCRP (logged)	CRP (mg/dL) (logged)
	0.076	LBDSGBSI	Globulin (g/L)
	0.072	LBDLYMNO	Lymphocyte number (1000 cells/uL)
	0.059	BMXBMI (logged)	Body Mass Index (kg/m2) (logged)
	0.058	LBDTIBSI	Total iron binding capacity (umol/L)
	0.053	LBXPLTSI	Platelet count (1000 cells/uL)
	0.042	LBDSUASI	Uric acid (umol/L)
	0.037	LBXLYPCT	Lymphocyte percent
	0.036	LBXMPSI	Mean platelet volume (fL)
	0.029	LBXCOT	Cotinine (ng/mL)
	0.024	LBXWBCSI (logged)	WBC count (1000 cells/uL) (logged)
	0.021	LBDSBUSI	Blood Urea Nitrogen (mmol/L)
	0.016	LBDSCRSI	Creatinine (umol/L)
	0.015	LBDSPHSI	Phosphorus (mmol/L)
	0.014	LBXSC3SI	Bicarbonate (mmol/L)
	0.012	fs1Score	Co-morbidity index
	0.011	SSBNP (logged)	NT-proBNP (pg/ml) (logged)
	0.006	LBDNENO	Segmented neutrophils number (1000 cell/uL)
	0.004	LBDBANO	Basophils number (1000 cells/uL)
	0.002	LBXSKSI	Potassium (mmol/L)
	-0.494	URXUMASI	Albumin urine (mg/L)
	-0.388	crAlbRat (logged)	Log Urine Albumin-to-Creatinine Ratio (mg/g) (logged)
	-0.227	LBXHGB	Hemoglobin (g/dL)
	-0.191	LBXHCT	Hematocrit
	-0.185	LBXPCT	Transferrin Saturation (%)
	-0.178	LBDIRNSI	Iron (umol/L)
	-0.173	LBXMCHSI	Mean cell hemoglobin (pg)
	-0.167	LBXMCVSI	Mean cell volume (fL)
	-0.142	LBDSTBSI	Bilirubin total (umol/L)
	-0.141	LBDSALSI	Albumin (g/L)
	-0.11	LBXSASSI (logged)	Aspartate Aminotransferase (AST) (U/L) (logged)
	-0.1	LBXSATSI (logged)	Alanine Aminotransferase (ALT) (U/L) (logged)
	-0.099	LBXMC	Mean Cell Hemoglobin Concentration (g/dL)
	-0.089	LBDFERSI (logged)	Ferritin (ug/L) (logged)
	-0.073	URXUCRSI (logged)	Creatinine urine (umol/L) (logged)
	-0.067	LBXRBCSI	Red blood cell count (million cells/uL)
	-0.063	BPXDAR	DBP average reported to examinee
	-0.06	LBXSNASI	Sodium (mmol/L)
	-0.057	LBDFOLSI (logged)	Folate serum (nmol/L) (logged)
	-0.052	LBXNEPCT	Segmented neutrophils percent
	-0.046	BPXSAR	SBP average reported to examinee
	-0.044	LBDSCASI	Calcium total (mmol/L)
	-0.023	LBXSCLSI	Chloride (mmol/L)
	-0.017	LBDSTPSI	Protein total (g/L)
	-0.016	LDLV	Low-Density Lipoprotein (mmol/L)
	-0.014	LBXBAPCT	Basophils percent
	-0.014	LBDSGLSI	Glucose (mmol/L)
	-0.013	BPXPLS	60 sec pulse (30 sec pulse X2)
	-0.012	LBXSLDSI (logged)	Lactate Dehydrogenase (LDH) (U/L) (logged)
	-0.009	LBDB12SI (logged)	Vitamin B12 serum (pmol/L) (logged)
	-0.004	LBXSAPSI (logged)	Alkaline Phosphatase (ALP) (IU/L) (logged)

PC6F	0.295	LBXWBCSI (logged)	WBC count (1000 cells/uL) (logged)
	0.293	LBDSBUSI	Blood Urea Nitrogen (mmol/L)
	0.29	LBDNENO	Segmented neutrophils number (1000 cell/uL)
	0.259	LBDSUASI	Uric acid (umol/L)
	0.249	LBDSCRSI	Creatinine (umol/L)
	0.23	LBDMONO	Monocyte number (1000 cells/uL)
	0.194	LBXHGB	Hemoglobin (g/dL)
	0.184	SSBNP (logged)	NT-proBNP (pg/ml) (logged)
	0.183	LBXNEPCT	Segmented neutrophils percent
	0.168	LBXHCT	Hematocrit
	0.133	LBXCRP (logged)	CRP (mg/dL) (logged)
	0.131	LBXSKSI	Potassium (mmol/L)
	0.119	LBXMCHSI	Mean cell hemoglobin (pg)
	0.113	LBXMCVSI	Mean cell volume (fL)
	0.111	LBDLYMNO	Lymphocyte number (1000 cells/uL)
	0.096	LBDSCASI	Calcium total (mmol/L)
	0.089	LBXCOT	Cotinine (ng/mL)
	0.085	LBXPLTSI	Platelet count (1000 cells/uL)
	0.083	LBDFERSI (logged)	Ferritin (ug/L) (logged)
	0.08	LBXRBCSI	Red blood cell count (million cells/uL)
	0.076	LBXMC	Mean Cell Hemoglobin Concentration (g/dL)
	0.06	BPXPLS	60 sec pulse (30 sec pulse X2)
	0.057	LBDEONO	Eosinophils number (1000 cells/uL)
	0.056	LBDSPHSI	Phosphorus (mmol/L)
	0.051	LDLV	Low-Density Lipoprotein (mmol/L)
	0.046	BMXBMI (logged)	Body Mass Index (kg/m2) (logged)
	0.041	LBXSAPSI (logged)	Alkaline Phosphatase (ALP) (IU/L) (logged)
	0.038	LBXPCT	Transferrin Saturation (%)
	0.036	LBDIRNSI	Iron (umol/L)
	0.034	LBDSGBSI	Globulin (g/L)
	0.019	fs2Score	Self-health index
	0.017	LBDSTPSI	Protein total (g/L)
	0.009	URXUCRSI (logged)	Creatinine urine (umol/L) (logged)
	0.003	LBXMPSI	Mean platelet volume (fL)
	0.003	fs1Score	Co-morbidity index
	0	LBDBANO	Basophils number (1000 cells/uL)
	-0.291	LBXSC3SI	Bicarbonate (mmol/L)
	-0.176	LBXLYPCT	Lymphocyte percent
	-0.176	LBDSGLSI	Glucose (mmol/L)
	-0.154	LBXBAPCT	Basophils percent
	-0.136	LBXSASSI (logged)	Aspartate Aminotransferase (AST) (U/L) (logged)
	-0.128	LBXGH	Glycohemoglobin (%)
	-0.115	URXUMASI	Albumin urine (mg/L)
	-0.112	LBXMOPCT	Monocyte percent
	-0.092	LBXSATSI (logged)	Alanine Aminotransferase (ALT) (U/L) (logged)
	-0.088	crAlbRat (logged)	Log Urine Albumin-to-Creatinine Ratio (mg/g) (logged)
	-0.085	BPXDAR	DBP average reported to examinee
	-0.079	LBDSTBSI	Bilirubin total (umol/L)
	-0.078	fs3Score	Healthcare use index
	-0.077	LBDB12SI (logged)	Vitamin B12 serum (pmol/L) (logged)
	-0.065	LBXRDW	Red cell distribution width (percent)
	-0.055	LBXSNASI	Sodium (mmol/L)
	-0.049	LBXEOPCT	Eosinophils percent
	-0.047	LBXSLDSI (logged)	Lactate Dehydrogenase (LDH) (U/L) (logged)
	-0.046	LBDFOLSI (logged)	Folate serum (nmol/L) (logged)
	-0.033	LBXSCLSI	Chloride (mmol/L)
	-0.025	LBDTIBSI	Total iron binding capacity (umol/L)
	-0.024	LBDSALSI	Albumin (g/L)
	-0.022	BPXSAR	SBP average reported to examinee

PC11F	0.539	BPXSAR	SBP average reported to examinee
	0.342	BPXDAR	DBP average reported to examinee
	0.222	fs2Score	Self-health index
	0.139	SSBNP (logged)	NT-proBNP (pg/ml) (logged)
	0.122	LBXMCHSI	Mean cell hemoglobin (pg)
	0.111	LBXMCVSI	Mean cell volume (fL)
	0.109	LBDSGBSI	Globulin (g/L)
	0.085	LBXMC	Mean Cell Hemoglobin Concentration (g/dL)
	0.08	LBDSTPSI	Protein total (g/L)
	0.061	LBXPCT	Transferrin Saturation (%)
	0.059	LBXLYPCT	Lymphocyte percent
	0.053	LBDFERSI (logged)	Ferritin (ug/L) (logged)
	0.05	LBXPLTSI	Platelet count (1000 cells/uL)
	0.046	LBDLYMNO	Lymphocyte number (1000 cells/uL)
	0.043	LBDIRNSI	Iron (umol/L)
	0.041	LBXCRP (logged)	CRP (mg/dL) (logged)
	0.037	LBXSLDSI (logged)	Lactate Dehydrogenase (LDH) (U/L) (logged)
	0.037	LBDSTBSI	Bilirubin total (umol/L)
	0.021	BMXBMI (logged)	Body Mass Index (kg/m2) (logged)
	0.021	crAlbRat (logged)	Log Urine Albumin-to-Creatinine Ratio (mg/g) (logged)
	0.019	BPXPLS	60 sec pulse (30 sec pulse X2)
	0.016	LBXSAPSI (logged)	Alkaline Phosphatase (ALP) (IU/L) (logged)
	0.013	LBDNENO	Segmented neutrophils number (1000 cell/uL)
	0.009	LBXWBCSI (logged)	WBC count (1000 cells/uL) (logged)
	0.004	fs1Score	Co-morbidity index
	0.003	LBXNEPCT	Segmented neutrophils percent
	0.002	LBXMOPCT	Monocyte percent
	-0.333	LBXEOPCT	Eosinophils percent
	-0.222	LBXSKSI	Potassium (mmol/L)
	-0.22	LBDEONO	Eosinophils number (1000 cells/uL)
	-0.216	LBDSBUSI	Blood Urea Nitrogen (mmol/L)
	-0.177	LBXBAPCT	Basophils percent
	-0.169	LBXRBCSI	Red blood cell count (million cells/uL)
	-0.13	LBXSCLSI	Chloride (mmol/L)
	-0.126	LBXSNASI	Sodium (mmol/L)
	-0.113	LBXMPSI	Mean platelet volume (fL)
	-0.11	LBXHCT	Hematocrit
	-0.105	LBXSATSI (logged)	Alanine Aminotransferase (ALT) (U/L) (logged)
	-0.095	LBXSASSI (logged)	Aspartate Aminotransferase (AST) (U/L) (logged)
	-0.092	URXUMASI	Albumin urine (mg/L)
	-0.09	LBXSC3SI	Bicarbonate (mmol/L)
	-0.085	URXUCRSI (logged)	Creatinine urine (umol/L) (logged)
	-0.083	LBXHGB	Hemoglobin (g/dL)
	-0.076	LBXGH	Glycohemoglobin (%)
	-0.066	LBDSPHSI	Phosphorus (mmol/L)
	-0.054	LBDTIBSI	Total iron binding capacity (umol/L)
	-0.052	LBDSCASI	Calcium total (mmol/L)
	-0.05	LBDFOLSI (logged)	Folate serum (nmol/L) (logged)
	-0.049	LBDSCRSI	Creatinine (umol/L)
	-0.046	LBDSALSI	Albumin (g/L)
	-0.038	LBXRDW	Red cell distribution width (percent)
	-0.024	LBDSGLSI	Glucose (mmol/L)
	-0.018	LBDSUASI	Uric acid (umol/L)
	-0.017	LBDB12SI (logged)	Vitamin B12 serum (pmol/L) (logged)
	-0.007	LBXCOT	Cotinine (ng/mL)
	-0.007	LBDBANO	Basophils number (1000 cells/uL)
	-0.007	fs3Score	Healthcare use index
	-0.007	LDLV	Low-Density Lipoprotein (mmol/L)
	-0.001	LBDMONO	Monocyte number (1000 cells/uL)

PC13F	0.427	LBXSC3SI	Bicarbonate (mmol/L)
	0.308	LBXSNASI	Sodium (mmol/L)
	0.284	LBDMONO	Monocyte number (1000 cells/uL)
	0.267	fs2Score	Self-health index
	0.238	LBXSCLSI	Chloride (mmol/L)
	0.184	LBDLYMNO	Lymphocyte number (1000 cells/uL)
	0.167	LBXSATSI (logged)	Alanine Aminotransferase (ALT) (U/L) (logged)
	0.156	LBXWBCSI (logged)	WBC count (1000 cells/uL) (logged)
	0.154	LBXSASSI (logged)	Aspartate Aminotransferase (AST) (U/L) (logged)
	0.13	SSBNP (logged)	NT-proBNP (pg/ml) (logged)
	0.121	LBXMOPCT	Monocyte percent
	0.092	LBXMCVSI	Mean cell volume (fL)
	0.089	LBXMCHSI	Mean cell hemoglobin (pg)
	0.086	LBDNENO	Segmented neutrophils number (1000 cell/uL)
	0.082	URXUCRSI (logged)	Creatinine urine (umol/L) (logged)
	0.074	LBDFERSI (logged)	Ferritin (ug/L) (logged)
	0.074	LBXCOT	Cotinine (ng/mL)
	0.067	LBXLYPCT	Lymphocyte percent
	0.063	LBXSAPSI (logged)	Alkaline Phosphatase (ALP) (IU/L) (logged)
	0.059	LBXSLDSI (logged)	Lactate Dehydrogenase (LDH) (U/L) (logged)
	0.05	LBXMPSI	Mean platelet volume (fL)
	0.043	LBXCRP (logged)	CRP (mg/dL) (logged)
	0.036	LBXGH	Glycohemoglobin (%)
	0.034	LBXMC	Mean Cell Hemoglobin Concentration (g/dL)
	0.029	URXUMASI	Albumin urine (mg/L)
	0.028	BMXBMI (logged)	Body Mass Index (kg/m2) (logged)
	0.028	LBDSPHSI	Phosphorus (mmol/L)
	0.012	LBDB12SI (logged)	Vitamin B12 serum (pmol/L) (logged)
	0.002	LBXHGB	Hemoglobin (g/dL)
	0	fs1Score	Co-morbidity index
	-0.273	LBDSTBSI	Bilirubin total (umol/L)
	-0.237	fs3Score	Healthcare use index
	-0.208	LBDSTPSI	Protein total (g/L)
	-0.134	LBDFOLSI (logged)	Folate serum (nmol/L) (logged)
	-0.129	LBDSGBSI	Globulin (g/L)
	-0.12	LBXEOPCT	Eosinophils percent
	-0.119	LBDSALSI	Albumin (g/L)
	-0.111	BPXDAR	DBP average reported to examinee
	-0.111	LBDSBUSI	Blood Urea Nitrogen (mmol/L)
	-0.108	LBDTIBSI	Total iron binding capacity (umol/L)
	-0.094	LBDSCASI	Calcium total (mmol/L)
	-0.072	LBXRBCSI	Red blood cell count (million cells/uL)
	-0.065	LBDIRNSI	Iron (umol/L)
	-0.065	LBXNEPCT	Segmented neutrophils percent
	-0.052	BPXPLS	60 sec pulse (30 sec pulse X2)
	-0.052	LBDSCRSI	Creatinine (umol/L)
	-0.045	LBDEONO	Eosinophils number (1000 cells/uL)
	-0.043	LBXBAPCT	Basophils percent
	-0.043	LBDSGLSI	Glucose (mmol/L)
	-0.038	LBXRDW	Red cell distribution width (percent)
	-0.021	LBDSUASI	Uric acid (umol/L)
	-0.019	crAlbRat (logged)	Log Urine Albumin-to-Creatinine Ratio (mg/g) (logged)
	-0.018	LBXPCT	Transferrin Saturation (%)
	-0.013	BPXSAR	SBP average reported to examinee
	-0.011	LBXHCT	Hematocrit
	-0.01	LBXSKSI	Potassium (mmol/L)
	-0.01	LDLV	Low-Density Lipoprotein (mmol/L)
	-0.008	LBXPLTSI	Platelet count (1000 cells/uL)
	-0.001	LBDBANO	Basophils number (1000 cells/uL)

PC20F	0.331	LBXPLTSI	Platelet count (1000 cells/uL)
	0.243	LBXRDW	Red cell distribution width (percent)
	0.233	fs2Score	Self-health index
	0.231	LBXSKSI	Potassium (mmol/L)
	0.225	SSBNP (logged)	NT-proBNP (pg/ml) (logged)
	0.18	LBXBAPCT	Basophils percent
	0.172	LBXPCT	Transferrin Saturation (%)
	0.17	LDLV	Low-Density Lipoprotein (mmol/L)
	0.165	LBDSCRSI	Creatinine (umol/L)
	0.157	LBDIRNSI	Iron (umol/L)
	0.136	LBXSAPSI (logged)	Alkaline Phosphatase (ALP) (IU/L) (logged)
	0.105	LBXSLDSI (logged)	Lactate Dehydrogenase (LDH) (U/L) (logged)
	0.09	LBXSASSI (logged)	Aspartate Aminotransferase (AST) (U/L) (logged)
	0.089	LBXCOT	Cotinine (ng/mL)
	0.088	LBXSC3SI	Bicarbonate (mmol/L)
	0.065	LBDSGLSI	Glucose (mmol/L)
	0.056	LBDSPHSI	Phosphorus (mmol/L)
	0.054	LBXLYPCT	Lymphocyte percent
	0.045	LBDSCASI	Calcium total (mmol/L)
	0.042	LBDSALSI	Albumin (g/L)
	0.03	LBDSTPSI	Protein total (g/L)
	0.027	LBDSTBSI	Bilirubin total (umol/L)
	0.024	LBXGH	Glycohemoglobin (%)
	0.021	BPXPLS	60 sec pulse (30 sec pulse X2)
	0.018	crAlbRat (logged)	Log Urine Albumin-to-Creatinine Ratio (mg/g) (logged)
	0.009	LBXMCVSI	Mean cell volume (fL)
	0.008	LBXEOPCT	Eosinophils percent
	0.005	LBDBANO	Basophils number (1000 cells/uL)
	0.003	LBDSGBSI	Globulin (g/L)
	-0.401	LBXMPSI	Mean platelet volume (fL)
	-0.212	LBDMONO	Monocyte number (1000 cells/uL)
	-0.207	fs3Score	Healthcare use index
	-0.183	LBDFOLSI (logged)	Folate serum (nmol/L) (logged)
	-0.174	LBXMOPCT	Monocyte percent
	-0.158	LBDB12SI (logged)	Vitamin B12 serum (pmol/L) (logged)
	-0.146	BPXDAR	DBP average reported to examinee
	-0.139	LBXSCLSI	Chloride (mmol/L)
	-0.126	BMXBMI (logged)	Body Mass Index (kg/m2) (logged)
	-0.106	BPXSAR	SBP average reported to examinee
	-0.097	LBXMC	Mean Cell Hemoglobin Concentration (g/dL)
	-0.09	LBXCRP (logged)	CRP (mg/dL) (logged)
	-0.08	LBDSBUSI	Blood Urea Nitrogen (mmol/L)
	-0.07	LBDFERSI (logged)	Ferritin (ug/L) (logged)
	-0.067	URXUMASI	Albumin urine (mg/L)
	-0.067	URXUCRSI (logged)	Creatinine urine (umol/L) (logged)
	-0.066	LBXSNASI	Sodium (mmol/L)
	-0.054	LBDNENO	Segmented neutrophils number (1000 cell/uL)
	-0.05	LBXWBCSI (logged)	WBC count (1000 cells/uL) (logged)
	-0.047	LBDSUASI	Uric acid (umol/L)
	-0.042	LBXSATSI (logged)	Alanine Aminotransferase (ALT) (U/L) (logged)
	-0.034	LBXHGB	Hemoglobin (g/dL)
	-0.026	LBDEONO	Eosinophils number (1000 cells/uL)
	-0.024	LBXNEPCT	Segmented neutrophils percent
	-0.024	LBXMCHSI	Mean cell hemoglobin (pg)
	-0.014	LBDTIBSI	Total iron binding capacity (umol/L)
	-0.01	LBXRBCSI	Red blood cell count (million cells/uL)
	-0.009	LBDLYMNO	Lymphocyte number (1000 cells/uL)
	-0.005	LBXHCT	Hematocrit
	-0.001	fs1Score	Co-morbidity index

PC22F	0.169	LBXEOPCT	Eosinophils percent
	0.154	LBXSLDSI (logged)	Lactate Dehydrogenase (LDH) (U/L) (logged)
	0.14	LBXSC3SI	Bicarbonate (mmol/L)
	0.122	LBDEONO	Eosinophils number (1000 cells/uL)
	0.105	LBXHGB	Hemoglobin (g/dL)
	0.105	fs3Score	Healthcare use index
	0.102	LBXLYPCT	Lymphocyte percent
	0.096	LBXMC	Mean Cell Hemoglobin Concentration (g/dL)
	0.09	LBXSATSI (logged)	Alanine Aminotransferase (ALT) (U/L) (logged)
	0.088	LBXSASSI (logged)	Aspartate Aminotransferase (AST) (U/L) (logged)
	0.076	LBXHCT	Hematocrit
	0.066	LBXGH	Glycohemoglobin (%)
	0.06	SSBNP (logged)	NT-proBNP (pg/ml) (logged)
	0.053	LBXMCHSI	Mean cell hemoglobin (pg)
	0.053	LBDSBUSI	Blood Urea Nitrogen (mmol/L)
	0.05	BMXBMI (logged)	Body Mass Index (kg/m2) (logged)
	0.049	LBXRBCSI	Red blood cell count (million cells/uL)
	0.048	crAlbRat (logged)	Log Urine Albumin-to-Creatinine Ratio (mg/g) (logged)
	0.042	LBDSPHSI	Phosphorus (mmol/L)
	0.039	LBDLYMNO	Lymphocyte number (1000 cells/uL)
	0.039	LBXSKSI	Potassium (mmol/L)
	0.035	LBXRDW	Red cell distribution width (percent)
	0.024	LBXMCVSI	Mean cell volume (fL)
	0.015	BPXSAR	SBP average reported to examinee
	0.015	BPXDAR	DBP average reported to examinee
	0.011	URXUMASI	Albumin urine (mg/L)
	0.011	LBXSAPSI (logged)	Alkaline Phosphatase (ALP) (IU/L) (logged)
	0.01	URXUCRSI (logged)	Creatinine urine (umol/L) (logged)
	0.01	LBXCOT	Cotinine (ng/mL)
	0.002	fs1Score	Co-morbidity index
	-0.64	LBXBAPCT	Basophils percent
	-0.312	LBDFOLSI (logged)	Folate serum (nmol/L) (logged)
	-0.241	fs2Score	Self-health index
	-0.184	LBXSCLSI	Chloride (mmol/L)
	-0.175	LBDMONO	Monocyte number (1000 cells/uL)
	-0.166	LBXSNASI	Sodium (mmol/L)
	-0.137	LBDSUASI	Uric acid (umol/L)
	-0.135	LBDB12SI (logged)	Vitamin B12 serum (pmol/L) (logged)
	-0.122	LBXMOPCT	Monocyte percent
	-0.118	LBDSTPSI	Protein total (g/L)
	-0.117	LBDSGLSI	Glucose (mmol/L)
	-0.11	LBXMPSI	Mean platelet volume (fL)
	-0.109	LBDSTBSI	Bilirubin total (umol/L)
	-0.099	LBDSALSI	Albumin (g/L)
	-0.092	LBDSCASI	Calcium total (mmol/L)
	-0.089	LBDIRNSI	Iron (umol/L)
	-0.084	LBXPCT	Transferrin Saturation (%)
	-0.076	LBDNENO	Segmented neutrophils number (1000 cell/uL)
	-0.068	LBXNEPCT	Segmented neutrophils percent
	-0.066	LDLV	Low-Density Lipoprotein (mmol/L)
	-0.065	LBXPLTSI	Platelet count (1000 cells/uL)
	-0.055	LBXWBCSI (logged)	WBC count (1000 cells/uL) (logged)
	-0.053	LBDSGBSI	Globulin (g/L)
	-0.039	BPXPLS	60 sec pulse (30 sec pulse X2)
	-0.025	LBDFERSI (logged)	Ferritin (ug/L) (logged)
	-0.022	LBDBANO	Basophils number (1000 cells/uL)
	-0.016	LBDTIBSI	Total iron binding capacity (umol/L)
	-0.006	LBDSCRSI	Creatinine (umol/L)
	-0.005	LBXCRP (logged)	CRP (mg/dL) (logged)

PC23F	0.346	LBDB12SI (logged)	Vitamin B12 serum (pmol/L) (logged)
	0.304	LBXSAPSI (logged)	Alkaline Phosphatase (ALP) (IU/L) (logged)
	0.251	LBXSKSI	Potassium (mmol/L)
	0.219	LBDSGBSI	Globulin (g/L)
	0.215	LBXCOT	Cotinine (ng/mL)
	0.157	LBXRDW	Red cell distribution width (percent)
	0.147	LBDFERSI (logged)	Ferritin (ug/L) (logged)
	0.141	LBXPCT	Transferrin Saturation (%)
	0.135	URXUCRSI (logged)	Creatinine urine (umol/L) (logged)
	0.122	LBXMPSI	Mean platelet volume (fL)
	0.122	LBDSTPSI	Protein total (g/L)
	0.12	fs3Score	Healthcare use index
	0.11	LBXCRP (logged)	CRP (mg/dL) (logged)
	0.105	LBXNEPCT	Segmented neutrophils percent
	0.099	LBDNENO	Segmented neutrophils number (1000 cell/uL)
	0.097	BPXDAR	DBP average reported to examinee
	0.089	LBXMCVSI	Mean cell volume (fL)
	0.089	LBXSC3SI	Bicarbonate (mmol/L)
	0.087	LBXSCLSI	Chloride (mmol/L)
	0.08	LDLV	Low-Density Lipoprotein (mmol/L)
	0.071	LBDSPHSI	Phosphorus (mmol/L)
	0.064	LBDIRNSI	Iron (umol/L)
	0.06	LBDSGLSI	Glucose (mmol/L)
	0.052	LBXBAPCT	Basophils percent
	0.047	LBXPLTSI	Platelet count (1000 cells/uL)
	0.045	LBXWBCSI (logged)	WBC count (1000 cells/uL) (logged)
	0.039	LBXMCHSI	Mean cell hemoglobin (pg)
	0.033	LBXSNASI	Sodium (mmol/L)
	0.032	LBDSTBSI	Bilirubin total (umol/L)
	0.024	URXUMASI	Albumin urine (mg/L)
	0.013	BMXBMI (logged)	Body Mass Index (kg/m2) (logged)
	0.005	LBDBANO	Basophils number (1000 cells/uL)
	0	fs1Score	Co-morbidity index
	-0.387	fs2Score	Self-health index
	-0.26	LBDSUASI	Uric acid (umol/L)
	-0.186	LBDTIBSI	Total iron binding capacity (umol/L)
	-0.165	LBDFOLSI (logged)	Folate serum (nmol/L) (logged)
	-0.151	LBDSALSI	Albumin (g/L)
	-0.118	BPXPLS	60 sec pulse (30 sec pulse X2)
	-0.113	crAlbRat (logged)	Log Urine Albumin-to-Creatinine Ratio (mg/g) (logged)
	-0.107	LBDSCRSI	Creatinine (umol/L)
	-0.106	LBXEOPCT	Eosinophils percent
	-0.102	LBXRBCSI	Red blood cell count (million cells/uL)
	-0.09	LBXMC	Mean Cell Hemoglobin Concentration (g/dL)
	-0.087	LBXMOPCT	Monocyte percent
	-0.08	LBXLYPCT	Lymphocyte percent
	-0.077	LBXHGB	Hemoglobin (g/dL)
	-0.07	LBXGH	Glycohemoglobin (%)
	-0.062	LBXSATSI (logged)	Alanine Aminotransferase (ALT) (U/L) (logged)
	-0.049	LBXHCT	Hematocrit
	-0.04	LBXSASSI (logged)	Aspartate Aminotransferase (AST) (U/L) (logged)
	-0.04	LBDSCASI	Calcium total (mmol/L)
	-0.031	LBDEONO	Eosinophils number (1000 cells/uL)
	-0.025	LBDSBUSI	Blood Urea Nitrogen (mmol/L)
	-0.018	LBDMONO	Monocyte number (1000 cells/uL)
	-0.012	LBXSLDSI (logged)	Lactate Dehydrogenase (LDH) (U/L) (logged)
	-0.01	LBDLYMNO	Lymphocyte number (1000 cells/uL)
	-0.007	BPXSAR	SBP average reported to examinee
	-0.005	SSBNP (logged)	NT-proBNP (pg/ml) (logged)

PC24F	0.455	LBDSUASI	Uric acid (umol/L)
	0.304	LBXCOT	Cotinine (ng/mL)
	0.282	LBXRDW	Red cell distribution width (percent)
	0.271	LBDB12SI (logged)	Vitamin B12 serum (pmol/L) (logged)
	0.192	LBXPCT	Transferrin Saturation (%)
	0.184	crAlbRat (logged)	Log Urine Albumin-to-Creatinine Ratio (mg/g) (logged)
	0.168	LBDIRNSI	Iron (umol/L)
	0.098	LBXMPSI	Mean platelet volume (fL)
	0.097	BPXPLS	60 sec pulse (30 sec pulse X2)
	0.091	LBDFOLSI (logged)	Folate serum (nmol/L) (logged)
	0.087	LBDSCRSI	Creatinine (umol/L)
	0.076	LBDSGLSI	Glucose (mmol/L)
	0.074	LBXEOPCT	Eosinophils percent
	0.064	LBDSCASI	Calcium total (mmol/L)
	0.049	LBDEONO	Eosinophils number (1000 cells/uL)
	0.039	LBDSALSI	Albumin (g/L)
	0.035	BPXDAR	DBP average reported to examinee
	0.033	LBXMOPCT	Monocyte percent
	0.029	LBXSNASI	Sodium (mmol/L)
	0.02	LBXSCLSI	Chloride (mmol/L)
	0.013	LBXSKSI	Potassium (mmol/L)
	0.011	LBDSPHSI	Phosphorus (mmol/L)
	0.01	LBXSC3SI	Bicarbonate (mmol/L)
	0.007	LBXMCVSI	Mean cell volume (fL)
	-0.342	LBDSBUSI	Blood Urea Nitrogen (mmol/L)
	-0.286	LBXBAPCT	Basophils percent
	-0.247	URXUCRSI (logged)	Creatinine urine (umol/L) (logged)
	-0.129	LBDSTBSI	Bilirubin total (umol/L)
	-0.119	LBDSGBSI	Globulin (g/L)
	-0.114	SSBNP (logged)	NT-proBNP (pg/ml) (logged)
	-0.094	BMXBMI (logged)	Body Mass Index (kg/m2) (logged)
	-0.094	LBDSTPSI	Protein total (g/L)
	-0.089	LBXSLDSI (logged)	Lactate Dehydrogenase (LDH) (U/L) (logged)
	-0.083	fs3Score	Healthcare use index
	-0.082	LBXMC	Mean Cell Hemoglobin Concentration (g/dL)
	-0.079	fs2Score	Self-health index
	-0.075	LBXPLTSI	Platelet count (1000 cells/uL)
	-0.073	URXUMASI	Albumin urine (mg/L)
	-0.072	LBXSAPSI (logged)	Alkaline Phosphatase (ALP) (IU/L) (logged)
	-0.07	BPXSAR	SBP average reported to examinee
	-0.058	LDLV	Low-Density Lipoprotein (mmol/L)
	-0.056	LBXSATSI (logged)	Alanine Aminotransferase (ALT) (U/L) (logged)
	-0.054	LBXSASSI (logged)	Aspartate Aminotransferase (AST) (U/L) (logged)
	-0.053	LBXHGB	Hemoglobin (g/dL)
	-0.038	LBXGH	Glycohemoglobin (%)
	-0.033	LBXCRP (logged)	CRP (mg/dL) (logged)
	-0.031	LBXHCT	Hematocrit
	-0.03	LBXRBCSI	Red blood cell count (million cells/uL)
	-0.029	LBDTIBSI	Total iron binding capacity (umol/L)
	-0.028	LBDNENO	Segmented neutrophils number (1000 cell/uL)
	-0.027	LBXWBCSI (logged)	WBC count (1000 cells/uL) (logged)
	-0.023	LBDFERSI (logged)	Ferritin (ug/L) (logged)
	-0.021	LBXMCHSI	Mean cell hemoglobin (pg)
	-0.017	LBDLYMNO	Lymphocyte number (1000 cells/uL)
	-0.017	LBDMONO	Monocyte number (1000 cells/uL)
	-0.009	LBDBANO	Basophils number (1000 cells/uL)
	-0.005	LBXLYPCT	Lymphocyte percent
	-0.003	LBXNEPCT	Segmented neutrophils percent
	-0.002	fs1Score	Co-morbidity index

PC28F	0.248	LBDB12SI (logged)	Vitamin B12 serum (pmol/L) (logged)
	0.239	LBDSGLSI	Glucose (mmol/L)
	0.221	LBXCOT	Cotinine (ng/mL)
	0.202	LBXSLDSI (logged)	Lactate Dehydrogenase (LDH) (U/L) (logged)
	0.195	SSBNP (logged)	NT-proBNP (pg/ml) (logged)
	0.183	LBDSCRSI	Creatinine (umol/L)
	0.182	URXUCRSI (logged)	Creatinine urine (umol/L) (logged)
	0.18	LBXMCVSI	Mean cell volume (fL)
	0.167	LBDTIBSI	Total iron binding capacity (umol/L)
	0.158	LBXHGB	Hemoglobin (g/dL)
	0.155	LBXRDW	Red cell distribution width (percent)
	0.154	LBXHCT	Hematocrit
	0.15	LBXMCHSI	Mean cell hemoglobin (pg)
	0.127	LBXSAPSI (logged)	Alkaline Phosphatase (ALP) (IU/L) (logged)
	0.105	LBXSNASI	Sodium (mmol/L)
	0.104	LBXBAPCT	Basophils percent
	0.078	fs3Score	Healthcare use index
	0.076	BPXDAR	DBP average reported to examinee
	0.069	BPXPLS	60 sec pulse (30 sec pulse X2)
	0.054	LBDSALSI	Albumin (g/L)
	0.047	URXUMASI	Albumin urine (mg/L)
	0.044	LBXEOPCT	Eosinophils percent
	0.042	LBDSBUSI	Blood Urea Nitrogen (mmol/L)
	0.04	LBXLYPCT	Lymphocyte percent
	0.031	LBXSCLSI	Chloride (mmol/L)
	0.021	LBXRBCSI	Red blood cell count (million cells/uL)
	0.02	LBDLYMNO	Lymphocyte number (1000 cells/uL)
	0.019	LBDEONO	Eosinophils number (1000 cells/uL)
	0.018	LBDSCASI	Calcium total (mmol/L)
	0.009	LBXMC	Mean Cell Hemoglobin Concentration (g/dL)
	0.002	LBDBANO	Basophils number (1000 cells/uL)
	0.002	LBXSASSI (logged)	Aspartate Aminotransferase (AST) (U/L) (logged)
	0.002	fs1Score	Co-morbidity index
	-0.502	LBXSKSI	Potassium (mmol/L)
	-0.178	LBDFERSI (logged)	Ferritin (ug/L) (logged)
	-0.172	LBXPCT	Transferrin Saturation (%)
	-0.169	crAlbRat (logged)	Log Urine Albumin-to-Creatinine Ratio (mg/g) (logged)
	-0.131	LBXGH	Glycohemoglobin (%)
	-0.12	LBXCRP (logged)	CRP (mg/dL) (logged)
	-0.115	LDLV	Low-Density Lipoprotein (mmol/L)
	-0.108	LBDIRNSI	Iron (umol/L)
	-0.107	BMXBMI (logged)	Body Mass Index (kg/m2) (logged)
	-0.107	LBXMPSI	Mean platelet volume (fL)
	-0.097	LBXSATSI (logged)	Alanine Aminotransferase (ALT) (U/L) (logged)
	-0.08	LBXSC3SI	Bicarbonate (mmol/L)
	-0.08	LBDSGBSI	Globulin (g/L)
	-0.079	BPXSAR	SBP average reported to examinee
	-0.057	fs2Score	Self-health index
	-0.054	LBDSUASI	Uric acid (umol/L)
	-0.044	LBDSTPSI	Protein total (g/L)
	-0.04	LBXNEPCT	Segmented neutrophils percent
	-0.036	LBDSTBSI	Bilirubin total (umol/L)
	-0.032	LBDMONO	Monocyte number (1000 cells/uL)
	-0.028	LBXMOPCT	Monocyte percent
	-0.025	LBDNENO	Segmented neutrophils number (1000 cell/uL)
	-0.023	LBDFOLSI (logged)	Folate serum (nmol/L) (logged)
	-0.014	LBDSPHSI	Phosphorus (mmol/L)
	-0.009	LBXWBCSI (logged)	WBC count (1000 cells/uL) (logged)
	-0.009	LBXPLTSI	Platelet count (1000 cells/uL)

PC31F	0.47	LDLV	Low-Density Lipoprotein (mmol/L)
	0.322	LBDSGLSI	Glucose (mmol/L)
	0.243	LBDFOLSI (logged)	Folate serum (nmol/L) (logged)
	0.124	LBDLYMNO	Lymphocyte number (1000 cells/uL)
	0.114	LBXRDW	Red cell distribution width (percent)
	0.105	crAlbRat (logged)	Log Urine Albumin-to-Creatinine Ratio (mg/g) (logged)
	0.093	LBXSASSI (logged)	Aspartate Aminotransferase (AST) (U/L) (logged)
	0.087	LBDEONO	Eosinophils number (1000 cells/uL)
	0.086	LBXLYPCT	Lymphocyte percent
	0.082	LBDFERSI (logged)	Ferritin (ug/L) (logged)
	0.071	LBXEOPCT	Eosinophils percent
	0.067	LBDSBUSI	Blood Urea Nitrogen (mmol/L)
	0.065	LBXSCLSI	Chloride (mmol/L)
	0.064	LBXCRP (logged)	CRP (mg/dL) (logged)
	0.064	LBDSALSI	Albumin (g/L)
	0.057	LBXWBCSI (logged)	WBC count (1000 cells/uL) (logged)
	0.053	LBDMONO	Monocyte number (1000 cells/uL)
	0.053	fs2Score	Self-health index
	0.047	BPXPLS	60 sec pulse (30 sec pulse X2)
	0.045	LBDSTPSI	Protein total (g/L)
	0.018	LBXMPSI	Mean platelet volume (fL)
	0.016	LBXSAPSI (logged)	Alkaline Phosphatase (ALP) (IU/L) (logged)
	0.014	LBDNENO	Segmented neutrophils number (1000 cell/uL)
	0.012	SSBNP (logged)	NT-proBNP (pg/ml) (logged)
	0.009	LBXRBCSI	Red blood cell count (million cells/uL)
	0.007	LBXPCT	Transferrin Saturation (%)
	0.005	LBXMOPCT	Monocyte percent
	0.005	LBXMCVSI	Mean cell volume (fL)
	0.004	LBXHCT	Hematocrit
	0.004	fs3Score	Healthcare use index
	0.002	LBDSGBSI	Globulin (g/L)
	-0.47	LBXGH	Glycohemoglobin (%)
	-0.21	LBDTIBSI	Total iron binding capacity (umol/L)
	-0.209	LBDSUASI	Uric acid (umol/L)
	-0.18	LBDSCASI	Calcium total (mmol/L)
	-0.177	LBDSPHSI	Phosphorus (mmol/L)
	-0.164	LBXBAPCT	Basophils percent
	-0.161	LBDB12SI (logged)	Vitamin B12 serum (pmol/L) (logged)
	-0.132	LBXPLTSI	Platelet count (1000 cells/uL)
	-0.114	URXUCRSI (logged)	Creatinine urine (umol/L) (logged)
	-0.086	LBXMC	Mean Cell Hemoglobin Concentration (g/dL)
	-0.085	LBDIRNSI	Iron (umol/L)
	-0.084	BPXSAR	SBP average reported to examinee
	-0.081	LBXSC3SI	Bicarbonate (mmol/L)
	-0.08	LBXNEPCT	Segmented neutrophils percent
	-0.07	LBXSNASI	Sodium (mmol/L)
	-0.07	LBXSKSI	Potassium (mmol/L)
	-0.061	LBDSTBSI	Bilirubin total (umol/L)
	-0.04	BPXDAR	DBP average reported to examinee
	-0.034	URXUMASI	Albumin urine (mg/L)
	-0.033	LBDSCRSI	Creatinine (umol/L)
	-0.026	LBXHGB	Hemoglobin (g/dL)
	-0.026	LBXMCHSI	Mean cell hemoglobin (pg)
	-0.026	LBXSLDSI (logged)	Lactate Dehydrogenase (LDH) (U/L) (logged)
	-0.021	LBXCOT	Cotinine (ng/mL)
	-0.011	BMXBMI (logged)	Body Mass Index (kg/m2) (logged)
	-0.003	LBDBANO	Basophils number (1000 cells/uL)
	-0.003	LBXSATSI (logged)	Alanine Aminotransferase (ALT) (U/L) (logged)
	-0.001	fs1Score	Co-morbidity index

PC32F	0.298	LBDFOLSI (logged)	Folate serum (nmol/L) (logged)
	0.294	LBXSAPSI (logged)	Alkaline Phosphatase (ALP) (IU/L) (logged)
	0.273	LBXCOT	Cotinine (ng/mL)
	0.25	LBXRDW	Red cell distribution width (percent)
	0.236	LBDSBUSI	Blood Urea Nitrogen (mmol/L)
	0.184	LBDSPHSI	Phosphorus (mmol/L)
	0.154	LBDFERSI (logged)	Ferritin (ug/L) (logged)
	0.106	BPXSAR	SBP average reported to examinee
	0.105	BPXPLS	60 sec pulse (30 sec pulse X2)
	0.08	LBXSNASI	Sodium (mmol/L)
	0.076	LBXGH	Glycohemoglobin (%)
	0.06	LBXPCT	Transferrin Saturation (%)
	0.056	LBXBAPCT	Basophils percent
	0.049	LBXNEPCT	Segmented neutrophils percent
	0.048	LBXMCHSI	Mean cell hemoglobin (pg)
	0.047	LBXMCVSI	Mean cell volume (fL)
	0.044	LBDSCASI	Calcium total (mmol/L)
	0.037	crAlbRat (logged)	Log Urine Albumin-to-Creatinine Ratio (mg/g) (logged)
	0.036	LBXEOPCT	Eosinophils percent
	0.031	LBXCRP (logged)	CRP (mg/dL) (logged)
	0.027	LBXMC	Mean Cell Hemoglobin Concentration (g/dL)
	0.019	fs2Score	Self-health index
	0.018	LBXSATSI (logged)	Alanine Aminotransferase (ALT) (U/L) (logged)
	0.017	LBDEONO	Eosinophils number (1000 cells/uL)
	0.014	LBXSASSI (logged)	Aspartate Aminotransferase (AST) (U/L) (logged)
	0.013	LBDSGBSI	Globulin (g/L)
	0.004	fs1Score	Co-morbidity index
	0.003	LBXSC3SI	Bicarbonate (mmol/L)
	0.001	LBXHGB	Hemoglobin (g/dL)
	0	LBDBANO	Basophils number (1000 cells/uL)
	-0.368	LBDB12SI (logged)	Vitamin B12 serum (pmol/L) (logged)
	-0.304	LDLV	Low-Density Lipoprotein (mmol/L)
	-0.263	SSBNP (logged)	NT-proBNP (pg/ml) (logged)
	-0.186	LBDTIBSI	Total iron binding capacity (umol/L)
	-0.173	LBXMPSI	Mean platelet volume (fL)
	-0.171	LBXSKSI	Potassium (mmol/L)
	-0.156	LBDSTBSI	Bilirubin total (umol/L)
	-0.14	LBXSLDSI (logged)	Lactate Dehydrogenase (LDH) (U/L) (logged)
	-0.119	LBDSCRSI	Creatinine (umol/L)
	-0.118	LBXPLTSI	Platelet count (1000 cells/uL)
	-0.113	LBDSUASI	Uric acid (umol/L)
	-0.107	LBDSGLSI	Glucose (mmol/L)
	-0.076	LBDLYMNO	Lymphocyte number (1000 cells/uL)
	-0.072	fs3Score	Healthcare use index
	-0.064	LBXLYPCT	Lymphocyte percent
	-0.059	URXUMASI	Albumin urine (mg/L)
	-0.058	LBDMONO	Monocyte number (1000 cells/uL)
	-0.048	BMXBMI (logged)	Body Mass Index (kg/m2) (logged)
	-0.047	LBXWBCSI (logged)	WBC count (1000 cells/uL) (logged)
	-0.046	LBXSCLSI	Chloride (mmol/L)
	-0.032	LBXRBCSI	Red blood cell count (million cells/uL)
	-0.032	LBDSALSI	Albumin (g/L)
	-0.025	LBDIRNSI	Iron (umol/L)
	-0.012	LBDNENO	Segmented neutrophils number (1000 cell/uL)
	-0.009	LBXHCT	Hematocrit
	-0.008	BPXDAR	DBP average reported to examinee
	-0.008	LBDSTPSI	Protein total (g/L)
	-0.004	URXUCRSI (logged)	Creatinine urine (umol/L) (logged)
	-0.001	LBXMOPCT	Monocyte percent

PC35F	0.382	SSBNP (logged)	NT-proBNP (pg/ml) (logged)
	0.372	LDLV	Low-Density Lipoprotein (mmol/L)
	0.293	LBDSPHSI	Phosphorus (mmol/L)
	0.266	BPXPLS	60 sec pulse (30 sec pulse X2)
	0.155	LBDTIBSI	Total iron binding capacity (umol/L)
	0.154	LBXGH	Glycohemoglobin (%)
	0.143	LBXCOT	Cotinine (ng/mL)
	0.127	BPXDAR	DBP average reported to examinee
	0.126	LBXSAPSI (logged)	Alkaline Phosphatase (ALP) (IU/L) (logged)
	0.124	LBDSUASI	Uric acid (umol/L)
	0.119	LBDIRNSI	Iron (umol/L)
	0.087	LBDSBUSI	Blood Urea Nitrogen (mmol/L)
	0.078	URXUCRSI (logged)	Creatinine urine (umol/L) (logged)
	0.075	LBDFOLSI (logged)	Folate serum (nmol/L) (logged)
	0.066	LBXMOPCT	Monocyte percent
	0.056	LBXPCT	Transferrin Saturation (%)
	0.048	URXUMASI	Albumin urine (mg/L)
	0.047	LBXCRP (logged)	CRP (mg/dL) (logged)
	0.029	LBDFERSI (logged)	Ferritin (ug/L) (logged)
	0.022	LBXEOPCT	Eosinophils percent
	0.021	LBXSLDSI (logged)	Lactate Dehydrogenase (LDH) (U/L) (logged)
	0.015	LBXMPSI	Mean platelet volume (fL)
	0.013	LBXSNASI	Sodium (mmol/L)
	0.003	LBDSGBSI	Globulin (g/L)
	0	LBXNEPCT	Segmented neutrophils percent
	0	LBXMC	Mean Cell Hemoglobin Concentration (g/dL)
	-0.396	LBDSCRSI	Creatinine (umol/L)
	-0.212	LBXRDW	Red cell distribution width (percent)
	-0.199	BPXSAR	SBP average reported to examinee
	-0.152	LBXSKSI	Potassium (mmol/L)
	-0.133	LBXPLTSI	Platelet count (1000 cells/uL)
	-0.128	BMXBMI (logged)	Body Mass Index (kg/m2) (logged)
	-0.122	LBDSGLSI	Glucose (mmol/L)
	-0.112	LBXMCVSI	Mean cell volume (fL)
	-0.108	LBXHGB	Hemoglobin (g/dL)
	-0.106	LBXHCT	Hematocrit
	-0.091	LBXMCHSI	Mean cell hemoglobin (pg)
	-0.085	LBXSCLSI	Chloride (mmol/L)
	-0.069	LBXBAPCT	Basophils percent
	-0.062	crAlbRat (logged)	Log Urine Albumin-to-Creatinine Ratio (mg/g) (logged)
	-0.061	LBDLYMNO	Lymphocyte number (1000 cells/uL)
	-0.06	LBXWBCSI (logged)	WBC count (1000 cells/uL) (logged)
	-0.05	LBDSTBSI	Bilirubin total (umol/L)
	-0.048	LBDSALSI	Albumin (g/L)
	-0.048	fs3Score	Healthcare use index
	-0.043	LBDNENO	Segmented neutrophils number (1000 cell/uL)
	-0.041	LBXSC3SI	Bicarbonate (mmol/L)
	-0.039	LBXSATSI (logged)	Alanine Aminotransferase (ALT) (U/L) (logged)
	-0.028	LBDMONO	Monocyte number (1000 cells/uL)
	-0.028	LBDSTPSI	Protein total (g/L)
	-0.028	fs2Score	Self-health index
	-0.022	LBDEONO	Eosinophils number (1000 cells/uL)
	-0.019	LBDSCASI	Calcium total (mmol/L)
	-0.018	LBXRBCSI	Red blood cell count (million cells/uL)
	-0.014	LBXLYPCT	Lymphocyte percent
	-0.014	LBXSASSI (logged)	Aspartate Aminotransferase (AST) (U/L) (logged)
	-0.007	fs1Score	Co-morbidity index
	-0.005	LBDB12SI (logged)	Vitamin B12 serum (pmol/L) (logged)
	-0.003	LBDBANO	Basophils number (1000 cells/uL)

PC37F	0.367	LBDSPHSI	Phosphorus (mmol/L)
	0.209	LBXMCVSI	Mean cell volume (fL)
	0.184	LBDSUASI	Uric acid (umol/L)
	0.141	LBXSLDSI (logged)	Lactate Dehydrogenase (LDH) (U/L) (logged)
	0.129	LBXHCT	Hematocrit
	0.117	LBDSTBSI	Bilirubin total (umol/L)
	0.09	LBXPLTSI	Platelet count (1000 cells/uL)
	0.076	LBDFERSI (logged)	Ferritin (ug/L) (logged)
	0.071	LBXSKSI	Potassium (mmol/L)
	0.064	LBDSGLSI	Glucose (mmol/L)
	0.063	BPXSAR	SBP average reported to examinee
	0.058	LBXCOT	Cotinine (ng/mL)
	0.045	LBDSBUSI	Blood Urea Nitrogen (mmol/L)
	0.044	crAlbRat (logged)	Log Urine Albumin-to-Creatinine Ratio (mg/g) (logged)
	0.032	LBXBAPCT	Basophils percent
	0.023	BPXPLS	60 sec pulse (30 sec pulse X2)
	0.021	LBXSATSI (logged)	Alanine Aminotransferase (ALT) (U/L) (logged)
	0.017	LBDLYMNO	Lymphocyte number (1000 cells/uL)
	0.01	LBXMOPCT	Monocyte percent
	0.01	LBXMPSI	Mean platelet volume (fL)
	0.009	LBXLYPCT	Lymphocyte percent
	0.008	fs2Score	Self-health index
	0.005	LBDNENO	Segmented neutrophils number (1000 cell/uL)
	0.005	LBDSTPSI	Protein total (g/L)
	0.004	LBDMONO	Monocyte number (1000 cells/uL)
	0.003	LBDSGBSI	Globulin (g/L)
	0.002	LBDBANO	Basophils number (1000 cells/uL)
	0.002	LBDSALSI	Albumin (g/L)
	-0.504	LBDSCASI	Calcium total (mmol/L)
	-0.501	LBXMC	Mean Cell Hemoglobin Concentration (g/dL)
	-0.214	LBXRDW	Red cell distribution width (percent)
	-0.141	LBXCRP (logged)	CRP (mg/dL) (logged)
	-0.135	LBXPCT	Transferrin Saturation (%)
	-0.131	SSBNP (logged)	NT-proBNP (pg/ml) (logged)
	-0.129	LBDIRNSI	Iron (umol/L)
	-0.129	LDLV	Low-Density Lipoprotein (mmol/L)
	-0.113	BMXBMI (logged)	Body Mass Index (kg/m2) (logged)
	-0.108	URXUCRSI (logged)	Creatinine urine (umol/L) (logged)
	-0.093	LBXGH	Glycohemoglobin (%)
	-0.08	LBDFOLSI (logged)	Folate serum (nmol/L) (logged)
	-0.076	LBDSCRSI	Creatinine (umol/L)
	-0.065	LBXSC3SI	Bicarbonate (mmol/L)
	-0.051	BPXDAR	DBP average reported to examinee
	-0.047	LBXEOPCT	Eosinophils percent
	-0.047	LBXSNASI	Sodium (mmol/L)
	-0.044	URXUMASI	Albumin urine (mg/L)
	-0.032	LBDB12SI (logged)	Vitamin B12 serum (pmol/L) (logged)
	-0.026	LBXHGB	Hemoglobin (g/dL)
	-0.019	LBXRBCSI	Red blood cell count (million cells/uL)
	-0.019	LBXSAPSI (logged)	Alkaline Phosphatase (ALP) (IU/L) (logged)
	-0.018	LBXWBCSI (logged)	WBC count (1000 cells/uL) (logged)
	-0.009	LBDEONO	Eosinophils number (1000 cells/uL)
	-0.009	LBXMCHSI	Mean cell hemoglobin (pg)
	-0.007	fs3Score	Healthcare use index
	-0.005	fs1Score	Co-morbidity index
	-0.004	LBXSCLSI	Chloride (mmol/L)
	-0.003	LBDTIBSI	Total iron binding capacity (umol/L)
	-0.003	LBXSASSI (logged)	Aspartate Aminotransferase (AST) (U/L) (logged)
	-0.001	LBXNEPCT	Segmented neutrophils percent

PC38F	0.47	LBXCOT	Cotinine (ng/mL)
	0.279	LBXSLDSI (logged)	Lactate Dehydrogenase (LDH) (U/L) (logged)
	0.195	LBXSKSI	Potassium (mmol/L)
	0.132	LBXMC	Mean Cell Hemoglobin Concentration (g/dL)
	0.128	LBDSGBSI	Globulin (g/L)
	0.11	LBDFERSI (logged)	Ferritin (ug/L) (logged)
	0.099	LBDSUASI	Uric acid (umol/L)
	0.092	LBXRBCSI	Red blood cell count (million cells/uL)
	0.075	LBXSCLSI	Chloride (mmol/L)
	0.073	LBDNENO	Segmented neutrophils number (1000 cell/uL)
	0.07	LBDSBUSI	Blood Urea Nitrogen (mmol/L)
	0.063	LBXBAPCT	Basophils percent
	0.063	LBDSTPSI	Protein total (g/L)
	0.057	URXUCRSI (logged)	Creatinine urine (umol/L) (logged)
	0.052	LBXSASSI (logged)	Aspartate Aminotransferase (AST) (U/L) (logged)
	0.051	BPXDAR	DBP average reported to examinee
	0.048	LBXWBCSI (logged)	WBC count (1000 cells/uL) (logged)
	0.046	LBDSGLSI	Glucose (mmol/L)
	0.043	fs2Score	Self-health index
	0.042	LBDFOLSI (logged)	Folate serum (nmol/L) (logged)
	0.041	LBDLYMNO	Lymphocyte number (1000 cells/uL)
	0.036	LBXNEPCT	Segmented neutrophils percent
	0.033	LBXSC3SI	Bicarbonate (mmol/L)
	0.028	LBDTIBSI	Total iron binding capacity (umol/L)
	0.01	LBXHGB	Hemoglobin (g/dL)
	0.008	URXUMASI	Albumin urine (mg/L)
	0.007	LBDEONO	Eosinophils number (1000 cells/uL)
	0.007	LBDBANO	Basophils number (1000 cells/uL)
	0.005	LBDSTBSI	Bilirubin total (umol/L)
	0.003	LBDMONO	Monocyte number (1000 cells/uL)
	-0.329	LBDSPHSI	Phosphorus (mmol/L)
	-0.266	LBXPLTSI	Platelet count (1000 cells/uL)
	-0.234	LBXMPSI	Mean platelet volume (fL)
	-0.234	LBXSAPSI (logged)	Alkaline Phosphatase (ALP) (IU/L) (logged)
	-0.184	BPXPLS	60 sec pulse (30 sec pulse X2)
	-0.181	LBXCRP (logged)	CRP (mg/dL) (logged)
	-0.18	LBXMCVSI	Mean cell volume (fL)
	-0.17	BMXBMI (logged)	Body Mass Index (kg/m2) (logged)
	-0.149	LBDSCRSI	Creatinine (umol/L)
	-0.14	LBXSNASI	Sodium (mmol/L)
	-0.137	LBXRDW	Red cell distribution width (percent)
	-0.122	LBXSATSI (logged)	Alanine Aminotransferase (ALT) (U/L) (logged)
	-0.102	LBXMCHSI	Mean cell hemoglobin (pg)
	-0.101	LBDSALSI	Albumin (g/L)
	-0.091	LBXEOPCT	Eosinophils percent
	-0.089	LDLV	Low-Density Lipoprotein (mmol/L)
	-0.078	LBXPCT	Transferrin Saturation (%)
	-0.068	fs3Score	Healthcare use index
	-0.065	LBDIRNSI	Iron (umol/L)
	-0.057	LBDSCASI	Calcium total (mmol/L)
	-0.04	LBXMOPCT	Monocyte percent
	-0.037	crAlbRat (logged)	Log Urine Albumin-to-Creatinine Ratio (mg/g) (logged)
	-0.032	LBXHCT	Hematocrit
	-0.032	SSBNP (logged)	NT-proBNP (pg/ml) (logged)
	-0.025	BPXSAR	SBP average reported to examinee
	-0.023	LBDB12SI (logged)	Vitamin B12 serum (pmol/L) (logged)
	-0.012	LBXLYPCT	Lymphocyte percent
	-0.002	LBXGH	Glycohemoglobin (%)
	-0.001	fs1Score	Co-morbidity index

PC39F	0.354	LBXCRP (logged)	CRP (mg/dL) (logged)
	0.232	LBXMCVSI	Mean cell volume (fL)
	0.23	LBDFERSI (logged)	Ferritin (ug/L) (logged)
	0.221	LBDTIBSI	Total iron binding capacity (umol/L)
	0.202	LBDSALSI	Albumin (g/L)
	0.162	LBDSCASI	Calcium total (mmol/L)
	0.108	LBXCOT	Cotinine (ng/mL)
	0.093	LBDSTBSI	Bilirubin total (umol/L)
	0.081	BMXBMI (logged)	Body Mass Index (kg/m2) (logged)
	0.08	LBXGH	Glycohemoglobin (%)
	0.076	LBDSBUSI	Blood Urea Nitrogen (mmol/L)
	0.072	LBXSAPSI (logged)	Alkaline Phosphatase (ALP) (IU/L) (logged)
	0.066	LBXSKSI	Potassium (mmol/L)
	0.064	SSBNP (logged)	NT-proBNP (pg/ml) (logged)
	0.061	BPXPLS	60 sec pulse (30 sec pulse X2)
	0.059	fs2Score	Self-health index
	0.051	URXUCRSI (logged)	Creatinine urine (umol/L) (logged)
	0.032	LBXMCHSI	Mean cell hemoglobin (pg)
	0.03	URXUMASI	Albumin urine (mg/L)
	0.03	LBXEOPCT	Eosinophils percent
	0.03	LBXHCT	Hematocrit
	0.023	BPXDAR	DBP average reported to examinee
	0.023	LBXPLTSI	Platelet count (1000 cells/uL)
	0.019	LBDFOLSI (logged)	Folate serum (nmol/L) (logged)
	0.016	LBXSLDSI (logged)	Lactate Dehydrogenase (LDH) (U/L) (logged)
	0.015	LBXSATSI (logged)	Alanine Aminotransferase (ALT) (U/L) (logged)
	0.014	LBXSC3SI	Bicarbonate (mmol/L)
	0.01	fs1Score	Co-morbidity index
	0.009	LBXLYPCT	Lymphocyte percent
	0.007	LBXMPSI	Mean platelet volume (fL)
	0.002	LBDIRNSI	Iron (umol/L)
	0.001	LBDEONO	Eosinophils number (1000 cells/uL)
	0	LBXMOPCT	Monocyte percent
	-0.49	LBDSPHSI	Phosphorus (mmol/L)
	-0.454	LBXMC	Mean Cell Hemoglobin Concentration (g/dL)
	-0.189	LBDSGBSI	Globulin (g/L)
	-0.124	LBXRBCSI	Red blood cell count (million cells/uL)
	-0.119	LBDSCRSI	Creatinine (umol/L)
	-0.109	LBXHGB	Hemoglobin (g/dL)
	-0.109	LBDSGLSI	Glucose (mmol/L)
	-0.106	LBXPCT	Transferrin Saturation (%)
	-0.086	LDLV	Low-Density Lipoprotein (mmol/L)
	-0.08	LBXBAPCT	Basophils percent
	-0.063	fs3Score	Healthcare use index
	-0.058	LBDSTPSI	Protein total (g/L)
	-0.056	BPXSAR	SBP average reported to examinee
	-0.055	LBDMONO	Monocyte number (1000 cells/uL)
	-0.047	LBDNENO	Segmented neutrophils number (1000 cell/uL)
	-0.038	LBDSUASI	Uric acid (umol/L)
	-0.037	LBXSCLSI	Chloride (mmol/L)
	-0.036	LBXSASSI (logged)	Aspartate Aminotransferase (AST) (U/L) (logged)
	-0.036	LBXSNASI	Sodium (mmol/L)
	-0.035	LBXWBCSI (logged)	WBC count (1000 cells/uL) (logged)
	-0.031	crAlbRat (logged)	Log Urine Albumin-to-Creatinine Ratio (mg/g) (logged)
	-0.021	LBDLYMNO	Lymphocyte number (1000 cells/uL)
	-0.008	LBDB12SI (logged)	Vitamin B12 serum (pmol/L) (logged)
	-0.008	LBXNEPCT	Segmented neutrophils percent
	-0.007	LBXRDW	Red cell distribution width (percent)
	-0.003	LBDBANO	Basophils number (1000 cells/uL)

Supplementary Table 4. PC weights in LinAge2.

PC (Male)	Weights	P-Value	PC (Female)	Weights	P-Value
Chronological age	0.0054220	< 2e-16	Chronological age	0.0074772	< 2e-16
PC1M	0.1798438	< 2e-16	PC1F	0.2607816	< 2e-16
PC2M	0.0341744	0.047737	PC2F	0.0551195	0.030598
PC5M	0.1333414	1.76e-10	PC4F	0.0656256	0.008313
PC6M	0.0693078	0.001074	PC6F	0.0805784	0.002204
PC8M	0.0492219	0.046424	PC11F	0.0788920	0.010210
PC11M	0.1156164	5.58e-05	PC13F	0.1761816	5.30e-07
PC15M	0.0681599	0.026055	PC20F	0.1916443	8.17e-06
PC16M	0.0739918	0.020443	PC22F	0.1376097	0.002230
PC17M	0.1031597	0.000752	PC23F	0.1006660	0.022460
PC19M	0.0778960	0.018922	PC24F	0.1192029	0.010945
PC24M	0.1012453	0.003816	PC28F	0.1469867	0.004360
PC25M	0.0769868	0.044519	PC31F	0.1109110	0.038749
PC27M	0.1032640	0.012580	PC32F	0.1897054	0.000791
PC31M	0.1326559	0.003628	PC35F	0.2200549	0.000213
PC33M	0.1198606	0.008850	PC37F	0.1413076	0.035837
PC36M	0.1011755	0.046204	PC38F	0.1253549	0.052444
PC42M	0.1711699	0.005489	PC39F	0.1463831	0.024302

Supplementary Table 5. PC interpretation in LinAge2.

PC (Male)	Causes of Death	Diseases and Sociological Factors	Mechanisms	Interventions / Management
PC1M	Early (within 0-5 years)<br>• Diabetes mellitus<br>• Cardiovascular disease<br>• Stroke<br>• Chronic lung disease<br>• Chronic kidney disease<br>• Cancer<br>• Alzheimer's disease<br>• Others<br><br>Late (within 10-20 years)<br>• Pneumonia<br>• Diabetes mellitus<br>• Cardiovascular disease<br>• Stroke<br>• Chronic lung disease<br>• Chronic kidney disease<br>• Cancer<br>• Alzheimer's disease<br>• Others	Cardiometabolic syndrome<br>• Obesity<br>• Hypertension<br>• Hypercholesterolemia<br>• Diabetes mellitus and insulin use<br>• Diabetic complications<br>• Cardiovascular disease, including congestive cardiac failure<br>• Stroke<br><br>Organ impairment<br>• Cognitive impairment<br>• Visual impairment<br>• Thyroid disease<br>• Chronic lung diseases (asthma, chronic bronchitis, emphysema)<br>• Chronic kidney disease<br>• Chronic liver disease<br>• Arthritis<br>• Osteoporosis<br>• Anemia<br><br>Cancer<br><br>Sociological factors<br>• Do not exercise (low vigorous activity, low moderate activity, and do less muscle strengthening)<br>• Alcohol use<br>• Low education<br>• Low income	• Vascular aging<br>• Metabolic aging<br>• Inflammation<br>• Neurodegeneration	• Screen for and consider appropriate management of cardiometabolic syndrome, organ impairment(s), and cancer<br>• Screen for and consider appropriate management of cognitive impairment (including thyroid disease)<br>• Manage obesity<br>• Increase exercise<br>• Reduce alcohol use<br>• Consider age-appropriate vaccinations
PC2M	Early (within 0-5 years)<br>• Diabetes mellitus<br>• Cardiovascular disease<br>• Cancer<br>• Others<br><br>Late (within 10-20 years)<br>• Pneumonia<br>• Cardiovascular disease<br>• Chronic lung disease<br>• Cancer<br>• Alzheimer's disease<br>• Others	Cardiovascular disease<br>• Cardiovascular disease, including congestive cardiac failure<br><br>Organ impairment<br>• Cognitive impairment<br>• Thyroid disease<br>• Emphysema<br>• Chronic kidney disease<br>• Arthritis<br>• Osteoporosis<br>• Anemia<br><br>Cancer<br><br>Sociological factors<br>• Thin<br>• High education<br>• High income	• Vascular aging<br>• Inflammation<br>• Neurodegeneration	• Screen for and consider appropriate management of cardiovascular disease, organ impairment(s), and cancer<br>• Screen for and consider appropriate management of cognitive impairment (including thyroid disease)<br>• Consider age-appropriate vaccinations
PC5M	Early (within 0-5 years)<br>• Cardiovascular disease<br>• Chronic lung disease<br>• Chronic kidney disease<br>• Cancer<br>• Others<br><br>Late (within 10-20 years)<br>• Cardiovascular disease<br>• Chronic lung disease<br>• Cancer<br>• Alzheimer's disease<br>• Others	Cardiovascular disease<br>• Hypertension<br>• Diabetic peripheral neuropathy<br>• Cardiovascular disease, including congestive cardiac failure<br>• Stroke<br><br>Organ impairment<br>• Cognitive impairment<br>• Visual impairment<br>• Chronic lung diseases (asthma, chronic bronchitis, emphysema)<br>• Arthritis<br>• Spine fracture<br><br>Sociological factors<br>• Thin<br>• Cigarette smoking<br>• Alcohol use<br>• Do not exercise (low vigorous activity and do less muscle strengthening)<br>• Low income	• Vascular aging (smoking-related)<br>• Lung disease-related<br>• Inflammation<br>• Neurodegeneration	• Screen for and consider appropriate management of cardiovascular disease, vascular risk factors, organ impairment(s), and cancer<br>• Screen for and consider appropriate management of cognitive impairment<br>• Quit smoking<br>• Reduce alcohol use<br>• Increase exercise<br>• Consider age-appropriate vaccinations

PC6M	Early (within 0-5 years)<br>• Others	Vascular risk factors<br>• Hypertension<br>• Diabetic peripheral neuropathy<br>• Peripheral arterial disease<br>• Diabetic foot ulcers<br><br>Organ impairment<br>• Cognitive impairment<br>• Thyroid disease<br>• Asthma<br>• Chronic liver disease<br>• Chronic kidney disease<br>• Anemia<br><br>Sociological factors<br>• Cigarette smoking<br>• Alcohol use<br>• Do not exercise (low vigorous activity, low moderate activity, and do less muscle strengthening)<br>• Low income	• Vascular aging (smoking-related)	• Screen for and consider appropriate management of vascular risk factors, especially diabetic complications of the lower limb<br>• Screen for and consider appropriate management of organ impairment(s)<br>• Quit smoking<br>• Reduce alcohol use<br>• Increase exercise
PC8M	Early (within 0-5 years)<br>• Others<br><br>Late (within 10-20 years)<br>• Others	Vascular risk factors<br>• Hypertension<br>• Hypercholesterolemia<br><br>Organ impairment<br>• Chronic liver disease<br>• Chronic kidney disease<br>• Anemia<br>• Wrist fracture<br>• Visual impairment<br><br>Sociological factors<br>• Obesity<br>• Do not exercise (low moderate activity)	• Vascular aging<br>• Inflammation	• Screen for and consider appropriate management of vascular risk factors<br>• Screen for and consider appropriate management of organ impairment(s)<br>• Manage obesity<br>• Increase exercise
PC11M	Early (within 0-5 years)<br>• Stroke<br>• Others	Organ impairment<br>• Emphysema<br>• Visual impairment<br><br>Sociological factors<br>• Thin<br>• Alcohol use<br>• Do not exercise (low vigorous and moderate activity)<br>• Low education<br>• Low income	• Indeterminate	• Screen for and consider appropriate management of organ impairment(s)<br>• Reduce alcohol use<br>• Increase exercise
PC15M	Early (within 0-5 years)<br>• Diabetes mellitus	Diabetic complications<br>• Diabetic peripheral neuropathy<br><br>Organ impairment<br>• Cognitive impairment<br>• Visual impairment<br>• Chronic bronchitis<br>• Emphysema<br><br>Sociological factors<br>• Thin<br>• Alcohol use<br>• Do not exercise (low vigorous activity, low moderate activity, and do less muscle strengthening)<br>• Low education<br>• Low income	• Inflammation	• Screen for and consider appropriate management of diabetes and its complications<br>• Screen for and consider appropriate management of organ impairment(s)<br>• Reduce alcohol use<br>• Increase exercise
PC16M	Early (within 0-5 years)<br>• Stroke<br>• Chronic lung disease<br>• Chronic kidney disease<br>• Cancer<br>• Others<br><br>Late (within 10-20 years)<br>• Pneumonia<br>• Diabetes mellitus<br>• Cardiovascular disease	Sociological factors<br>• Thin<br>• Cigarette smoking<br>• Alcohol use<br>• Do not exercise (low vigorous activity and sedentary)<br>• Low income	• Smoking-related	• Screen for and consider appropriate management of smoking-related diseases (if smoker)<br>• Quit smoking<br>• Reduce alcohol use<br>• Increase exercise<br>• Consider age-appropriate vaccinations

	• Chronic kidney disease<br>• Cancer<br>• Others			
PC17M	Late (within 10-20 years)<br>• Diabetes mellitus	Vascular risk factors<br>• Hypertension<br><br>Sociological factors<br>• Do not exercise (less muscle strengthening)	• Indeterminate	• Screen for and consider appropriate management of vascular risk factors<br>• Increase exercise
PC19M	Early (within 0-5 years)<br>• Diabetes mellitus<br>• Stroke<br>• Others<br><br>Late (within 10-20 years)<br>• Alzheimer's disease<br>• Cancer	Cardiovascular disease<br>• Diabetes mellitus<br>• Ischemic heart disease<br><br>Organ impairment<br>• Thyroid disease<br><br>Sociological factors<br>• Thin<br>• Do not exercise (low vigorous activity)<br>• Low education<br>• Low income	• Vascular aging<br>• Neurodegeneration	• Screen for and consider appropriate management of cardiovascular disease and vascular risk factors<br>• Screen for and consider appropriate management of cognitive impairment (including thyroid disease)<br>• Screen for and consider appropriate management of cancer<br>• Increase exercise
PC24M	• None	Sociological factors<br>• Cigarette smoking	• Indeterminate	• Quit smoking
PC25M	Early (within 0-5 years)<br>• Cancer<br><br>Late (within 10-20 years)<br>• Others	Cardiovascular disease<br>• Hypertension<br>• Hyperlipidemia<br>• Cardiovascular disease, including congestive cardiac failure<br>• Stroke<br><br>Sociological factors<br>• Obesity<br>• Low education	• Vascular aging<br>• Inflammation	• Screen for and consider appropriate management of cardiovascular disease, vascular risk factors, and cancer<br>• Manage obesity
PC27M	Late (within 10-20 years)<br>• Chronic lung disease<br>• Cancer	Sociological factors<br>• Do not exercise (low moderate activity and sedentary)	• Indeterminate	• Screen for and consider appropriate management of chronic lung disease and cancer<br>• Increase exercise<br>• Consider age-appropriate vaccinations
PC31M	Early (within 0-5 years)<br>• Cardiovascular disease<br><br>Late (within 10-20 years)<br>• Cardiovascular disease	Cardiovascular disease<br>• Diabetes mellitus<br>• Cardiovascular disease, including congestive cardiac failure<br><br>Organ impairment<br>• Visual impairment<br><br>Cancer<br><br>Sociological factors<br>• Obesity<br>• Cigarette smoking<br>• Do not exercise (low vigorous activity)<br>• Low income	• Vascular aging (smoking-related)<br>• Cardiac disease-related	• Screen for and consider appropriate management of diabetes and cardiovascular disease<br>• Screen for and consider appropriate management of visual impairment and cancer<br>• Manage obesity<br>• Quit smoking<br>• Increase exercise
PC33M	• None	Cardiovascular disease<br>• Angina<br><br>Sociological factors<br>• High education	• Inflammation	• Screen for and consider appropriate management of cardiovascular disease
PC36M	Early (within 0-5 years)<br>• Diabetes mellitus	Vascular risk factors<br>• Diabetes mellitus<br><br>Sociological factors<br>• Cigarette smoking	• Indeterminate	• Screen for and consider appropriate management of diabetes<br>• Quit smoking

PC42M	• None	Vascular disease<br>• Peripheral arterial disease<br><br>Organ impairment<br>• Thyroid disease<br><br>Sociological factors<br>• Do not exercise (low vigorous and moderate activity)	• Indeterminate	• Screen for and consider appropriate management of peripheral arterial disease and thyroid disease<br>• Increase exercise
**PC (Female)**	**Causes of Death**	**Diseases and Sociological Factors**	**Mechanisms**	**Interventions / Management**
PC1F	Early (within 0-5 years)<br>• Pneumonia<br>• Cardiovascular disease<br>• Stroke<br>• Chronic lung disease<br>• Cancer<br>• Others<br><br>Late (within 10-20 years)<br>• Pneumonia<br>• Diabetes mellitus<br>• Cardiovascular disease<br>• Stroke<br>• Chronic lung disease<br>• Chronic kidney disease<br>• Cancer<br>• Alzheimer's disease<br>• Others	Cardiometabolic syndrome<br>• Obesity<br>• Hypertension<br>• Hypercholesterolemia<br>• Diabetes mellitus and insulin use<br>• Diabetic complications<br>• Cardiovascular disease, including congestive cardiac failure<br>• Stroke<br><br>Organ impairment<br>• Cognitive impairment<br>• Visual impairment<br>• Chronic lung diseases (asthma, chronic bronchitis, emphysema)<br>• Chronic kidney disease<br>• Chronic liver disease<br>• Arthritis<br>• Osteoporosis<br>• Anemia<br><br>Cancer<br><br>Falls<br><br>Sociological factors<br>• Do not exercise (low vigorous activity, low moderate activity, and do less muscle strengthening)<br>• Alcohol use<br>• Low education<br>• Low income	• Vascular aging<br>• Metabolic aging<br>• Inflammation<br>• Neurodegeneration	• Screen for and consider appropriate management of cardiometabolic syndrome, organ impairment(s), and cancer<br>• Screen for and consider appropriate management of geriatric syndromes (cognitive impairment, falls, osteoporosis)<br>• Manage obesity<br>• Increase exercise<br>• Reduce alcohol use<br>• Consider age-appropriate vaccinations
PC2F	Early (within 0-5 years)<br>• Cardiovascular disease<br>• Stroke<br>• Chronic lung disease<br>• Cancer<br>• Others<br><br>Late (within 10-20 years)<br>• Pneumonia<br>• Diabetes mellitus<br>• Cardiovascular disease<br>• Stroke<br>• Chronic lung disease<br>• Chronic kidney disease<br>• Cancer<br>• Alzheimer's disease<br>• Others	Cardiovascular disease<br>• Hypertension<br>• Hypercholesterolemia<br>• Diabetic foot ulcers<br>• Diabetic retinopathy<br>• Cardiovascular disease, including congestive cardiac failure<br>• Stroke<br><br>Organ impairment<br>• Cognitive impairment<br>• Thyroid disease<br>• Chronic bronchitis<br>• Emphysema<br>• Chronic liver disease<br>• Arthritis<br>• Osteoporosis<br>• Hip fracture<br>• Spine fracture<br><br>Cancer<br><br>Falls<br><br>Sociological factors<br>• Thin<br>• Do not exercise (sedentary)<br>• High education<br>• High income	• Vascular aging<br>• Neurodegeneration	• Screen for and consider appropriate management of cardiovascular disease, vascular risk factors, organ impairment(s), and cancer<br>• Screen for and consider appropriate management of geriatric syndromes (cognitive impairment, falls, osteoporosis)<br>• Increase exercise<br>• Consider age-appropriate vaccinations
PC4F	Early (within 0-5 years)<br>• Chronic lung disease<br><br>Late (within 10-20 years)	Cardiovascular disease<br>• Hypertension<br>• Diabetes mellitus and insulin use<br>• Diabetic peripheral neuropathy	• Vascular aging<br>• Inflammation	• Screen for and consider appropriate management of cardiovascular disease, vascular risk

	• Cardiovascular disease<br>• Chronic kidney disease	• Cardiovascular disease, including congestive cardiac failure<br>• Stroke<br><br><u>Organ impairment</u><br>• Cognitive impairment<br>• Visual impairment<br>• Asthma<br>• Chronic bronchitis<br>• Arthritis<br>• Osteoporosis<br>• Hip fracture<br>• Anemia<br><br><u>Cancer</u><br><br><u>Falls</u><br><br><u>Sociological factors</u><br>• Obesity<br>• Do not exercise (low vigorous and moderate activity)<br>• Low income		factors, organ impairment(s), and cancer<br>• Screen for and consider appropriate management of geriatric syndromes (cognitive impairment, falls, osteoporosis)<br>• Manage obesity<br>• Increase exercise<br>• Consider age-appropriate vaccinations
PC6F	<u>Early (within 0-5 years)</u><br>• Pneumonia<br>• Cardiovascular disease<br>• Chronic lung disease<br>• Cancer<br>• Others<br><br><u>Late (within 10-20 years)</u><br>• Cardiovascular disease<br>• Chronic lung disease<br>• Cancer<br>• Others	<u>Cardiovascular disease</u><br>• Hypertension<br>• Hypercholesterolemia<br>• Diabetic peripheral neuropathy<br>• Cardiovascular disease, including congestive cardiac failure<br><br><u>Organ impairment</u><br>• Thyroid disease<br>• Chronic bronchitis<br>• Chronic kidney disease<br>• Arthritis<br>• Osteoporosis<br>• Hip fracture<br><br><u>Sociological factors</u><br>• Obesity<br>• Cigarette smoking<br>• Alcohol use<br>• Do not exercise (low vigorous activity, low moderate activity, and do less muscle strengthening)	• Vascular aging (smoking-related)<br>• Inflammation	• Screen for and consider appropriate management of cardiovascular disease, vascular risk factors, and organ impairment(s)<br>• Manage obesity<br>• Quit smoking<br>• Reduce alcohol use<br>• Increase exercise<br>• Consider age-appropriate vaccinations
PC11F	<u>Early (within 0-5 years)</u><br>• Cardiovascular disease<br><br><u>Late (within 10-20 years)</u><br>• Others	<u>Cardiovascular disease</u><br>• Hypertension<br>• Diabetic retinopathy<br>• Diabetic peripheral neuropathy<br>• Peripheral arterial disease<br>• Ischemic heart disease<br>• Congestive cardiac failure<br>• Stroke<br><br><u>Organ impairment</u><br>• Cognitive impairment<br>• Arthritis<br><br><u>Sociological factors</u><br>• Alcohol use<br>• Do not exercise (low vigorous and moderate activity)<br>• Low education<br>• Low income	• Vascular aging	• Screen for and consider appropriate management of cardiovascular disease, vascular risk factors, diabetic complications, cognitive impairment, and arthritis<br>• Reduce alcohol use<br>• Increase exercise
PC13F	<u>Early (within 0-5 years)</u><br>• Cardiovascular disease<br>• Stroke<br>• Chronic lung disease<br><br><u>Late (within 10-20 years)</u><br>• Cardiovascular disease<br>• Cancer	<u>Cardiovascular disease</u><br>• Diabetic peripheral neuropathy<br>• Angina<br>• Acute myocardial infarction<br>• Stroke<br><br><u>Organ impairment</u><br>• Cognitive impairment<br>• Visual impairment<br>• Emphysema<br><br><u>Sociological factors</u><br>• Obesity	• Vascular aging	• Screen for and consider appropriate management of cardiovascular disease, vascular risk factors, cognitive impairment, visual impairment, chronic lung disease, and cancer<br>• Manage obesity<br>• Reduce alcohol use<br>• Increase exercise

		• Alcohol use<br>• Do not exercise (low vigorous activity, low moderate activity, and do less muscle strengthening)<br>• Low education<br>• Low income		• Consider age-appropriate vaccinations
PC20F	Early (within 0-5 years)<br>• Pneumonia<br>• Cardiovascular disease<br>• Others<br><br>Late (within 10-20 years)<br>• Chronic lung disease<br>• Others	Cardiovascular disease<br>• Hypercholesterolemia<br>• Angina<br>• Congestive cardiac failure<br>• Stroke<br><br>Organ impairment<br>• Visual impairment<br>• Emphysema<br>• Spine fracture<br>• Anemia<br><br>Sociological factors<br>• Thin<br>• Do not exercise (low vigorous activity, low moderate activity, and do less muscle strengthening)<br>• Low income	• Vascular aging	• Screen for and consider appropriate management of cardiovascular disease, vascular risk factors, and organ impairment(s) especially chronic lung disease<br>• Increase exercise<br>• Consider age-appropriate vaccinations
PC22F	Early (within 0-5 years)<br>• Others<br><br>Late (within 10-20 years)<br>• Chronic kidney disease<br>• Others	Cardiovascular disease<br>• Hypertension<br>• Insulin use<br>• Congestive cardiac failure<br><br>Organ impairment<br>• Asthma<br><br>Sociological factors<br>• Obesity	• Indeterminate	• Screen for and consider appropriate management of vascular risk factors, congestive cardiac failure, chronic kidney disease, and asthma<br>• Manage obesity
PC23F	Early (within 0-5 years)<br>• Cardiovascular disease<br>• Others<br><br>Late (within 10-20 years)<br>• Pneumonia<br>• Chronic lung disease<br>• Chronic kidney disease<br>• Cancer	Vascular risk factors<br>• Diabetic foot ulcers<br><br>Organ impairment<br>• Anemia<br><br>Sociological factors<br>• Thin<br>• Cigarette smoking<br>• Alcohol use	• Inflammation	• Screen for and consider appropriate management of vascular risk factors, diabetic complications, and anemia<br>• Quit smoking<br>• Reduce alcohol use
PC24F	Late (within 10-20 years)<br>• Chronic lung disease<br>• Cancer<br>• Alzheimer's disease<br>• Others	Sociological factors<br>• Thin<br>• Cigarette smoking<br>• Alcohol use	• Indeterminate	• Quit smoking<br>• Reduce alcohol use
PC28F	Early (within 0-5 years)<br>• Cardiovascular disease<br>• Cancer<br><br>Late (within 10-20 years)<br>• Others	Cardiovascular disease<br>• Diabetic retinopathy<br>• Diabetic peripheral neuropathy<br>• Acute myocardial infarction<br><br>Sociological factors<br>• Thin<br>• High income	• Indeterminate	• Screen for and consider appropriate management of cardiovascular disease, vascular risk factors, diabetic complications, and cancer
PC31F	Early (within 0-5 years)<br>• Cardiovascular disease<br>• Chronic lung disease<br><br>Late (within 10-20 years)<br>• Chronic lung disease<br>• Chronic kidney disease<br>• Cancer<br>• Others	Vascular risk factors<br>• Hypercholesterolemia<br><br>Organ impairment<br>• Cognitive impairment<br>• Emphysema<br>• Arthritis<br>• Hip fracture<br>• Anemia<br><br>Sociological factors<br>• Thin<br>• Low income	• Indeterminate	• Screen for and consider appropriate management of vascular risk factors and organ impairment(s) especially chronic lung disease<br>• Consider age-appropriate vaccinations
PC32F	Early (within 0-5 years)<br>• Stroke<br>• Others	Vascular risk factors<br>• Hypertension	• Indeterminate	• Screen for and consider appropriate management of

	Late (within 10-20 years)<br>• Diabetes mellitus<br>• Cancer<br>• Others	Organ impairment<br>• Chronic bronchitis<br>• Chronic kidney disease<br>• Arthritis<br><br>Sociological factors<br>• Cigarette smoking<br>• Alcohol use<br>• Do not exercise (low moderate activity and do less muscle strengthening)<br>• Low education		vascular risk factors and organ impairment(s)<br>• Quit smoking<br>• Reduce alcohol use<br>• Increase exercise
PC35F	Early (within 0-5 years)<br>• Cardiovascular disease<br>• Chronic lung disease<br>• Cancer<br>• Others<br><br>Late (within 10-20 years)<br>• Pneumonia<br>• Cardiovascular disease<br>• Chronic lung disease<br>• Alzheimer's disease	Cardiovascular disease<br>• Hypercholesterolemia<br>• Congestive cardiac failure<br><br>Sociological factors<br>• Thin<br>• Do not exercise (low vigorous activity, low moderate activity, and do less muscle strengthening)	• Vascular aging	• Screen for and consider appropriate management of cardiovascular disease, vascular risk factors, chronic lung disease, and cancer<br>• Increase exercise<br>• Consider age-appropriate vaccinations
PC37F	Early (within 0-5 years)<br>• Cardiovascular disease<br>• Cancer<br><br>Late (within 10-20 years)<br>• Cancer<br>• Others	Organ impairment<br>• Visual impairment	• Cancer-related	• Screen for and consider appropriate management of cancer, cardiovascular disease, and visual impairment
PC38F	• None	Organ impairment<br>• Emphysema<br><br>Sociological factors<br>• Thin<br>• Cigarette smoking<br>• Low income	• Smoking-related	• Screen for and consider appropriate management of emphysema<br>• Quit smoking
PC39F	Late (within 10-20 years)<br>• Pneumonia<br>• Cardiovascular disease<br>• Others	Vascular risk factors<br>• Hypertension<br><br>Organ impairment<br>• Chronic lung diseases (asthma, chronic bronchitis, emphysema)<br>• Arthritis<br><br>Sociological factors<br>• Obesity<br>• Alcohol use<br>• Do not exercise (low vigorous activity)<br>• Low education<br>• Low income	• Lung disease-related<br>• Inflammation	• Screen for and consider appropriate management of vascular risk factors, chronic lung disease, and arthritis<br>• Manage obesity<br>• Reduce alcohol use<br>• Increase exercise<br>• Consider age-appropriate vaccinations
